# TOP2B Is Required to Maintain the Adrenergic Neural Phenotype and for ATRA-Induced Differentiation of SH-SY5Y Neuroblastoma Cells

**DOI:** 10.1007/s12035-022-02949-6

**Published:** 2022-07-13

**Authors:** Mushtaq M. Khazeem, John W. Casement, George Schlossmacher, Niall S. Kenneth, Nielda K. Sumbung, Janice Yuen Tung Chan, Jade F. McGow, Ian G. Cowell, Caroline A. Austin

**Affiliations:** 1 The Faculty of Medical Sciences, Biosciences Institute, Newcastle University, Newcastle upon Tyne, NE2 4HH UK; 2 National Center of Hematology, Mustansiriyah University, Baghdad, Iraq; 3 Bioinformatics Support Unit, The Faculty of Medical Sciences, Newcastle University, Newcastle upon Tyne, NE2 4HH UK; 4 Institute of Systems, Molecular and Integrative Biology, University of Liverpool, Liverpool, L69 7ZB UK

**Keywords:** Neuroblastoma, Topoisomerase, TOP2B, SH-SY5Y, Retinoic acid, Differential gene expression

## Abstract

The neuroblastoma cell line SH-SY5Y is widely used to study retinoic acid (RA)-induced gene expression and differentiation and as a tool to study neurodegenerative disorders. SH-SY5Y cells predominantly exhibit adrenergic neuronal properties, but they can also exist in an epigenetically interconvertible alternative state with more mesenchymal characteristics; as a result, these cells can be used to study gene regulation circuitry controlling neuroblastoma phenotype. Using a combination of pharmacological inhibition and targeted gene inactivation, we have probed the requirement for DNA topoisomerase IIB (TOP2B) in RA-induced gene expression and differentiation and in the balance between adrenergic neuronal versus mesenchymal transcription programmes. We found that expression of many, but not all genes that are rapidly induced by ATRA in SH-SY5Y cells was significantly reduced in the TOP2B null cells; these genes include *BCL2*, *CYP26A1*, *CRABP2*, and *NTRK2*. Comparing gene expression profiles in wild-type versus TOP2B null cells, we found that long genes and genes expressed at a high level in WT SH-SY5Y cells were disproportionately dependent on TOP2B. Notably, TOP2B null SH-SY5Y cells upregulated mesenchymal markers vimentin (*VIM*) and fibronectin (*FN1*) and components of the NOTCH signalling pathway. Enrichment analysis and comparison with the transcription profiles of other neuroblastoma-derived cell lines supported the conclusion that TOP2B is required to fully maintain the adrenergic neural-like transcriptional signature of SH-SY5Y cells and to suppress the alternative mesenchymal epithelial-like epigenetic state.

**Supplementary Information:**

The online version contains supplementary material available at 10.1007/s12035-022-02949-6.

## Introduction

DNA topoisomerases are essential nuclear enzymes that are required for DNA decatenation, unknotting, and modulation of DNA topology. These transactions are achieved via transient DNA cleavage and passage of a second DNA segment through the gap before religation [1]. Type II DNA topoisomerases including vertebrate TOP2A and TOP2B can cleave both DNA strands allowing a second DNA duplex to pass through the enzyme-bound gap [2, 3]. Topoisomerases are required for efficient transcription where they regulate negative superhelical torsion behind, and positive torsion ahead of an elongating polymerase, as described in the twin domain model [2, 4, 5]. Transcription and topoisomerase activity may also work in concert to generate large-scale genomic topological organisation as TOP2B partially colocalises with CTCF/cohesin at the boundaries of chromosome topological associating domains (TADS) and loops [6, 7].

The vertebrate type II topoisomerases TOP2A and TOP2B have non-identical physiological roles. TOP2A is essential for cell proliferation, is abundantly expressed in proliferating cells, and is downregulated as cells terminally differentiate. In contrast, TOP2B is expressed in both proliferating and differentiated cells such as neurons and cardiomyocytes [8–13]. TOP2B null cell lines can be grown in culture, but TOP2B is essential in vivo [14]. TOP2B/Top2b nulls and hypomorphs exhibit defects in neural and B cell differentiation and function [9, 15] through mechanisms that appear to involve misregulation of specific sets of genes. TOP2B has been reported to regulate expression of long genes associated with autism in human and mouse neurons [16, 17] and to be necessary for normal neurite outgrowth in a number of studies including cultured rat cerebellar and cortical neurons and PC12 cells stimulated by neurotrophic growth factor (NGF) [18], mouse dopaminergic neurons in ventral mesencephalic mouse primary culture [19], and human mesenchymal stem cell-derived neurons [20, 21]. Top2b is also required for normal development in mouse and zebrafish [22, 23].

Various studies have demonstrated the importance of TOP2B for the expression of genes involved in neuronal development and maintenance. For instance, Top2b inhibition in cultured rat neurons reduced expression of amphiphysin I, an essential gene for neuronal function [24], and Top2b deletion reduced reelin expression in mouse embryos [25]. Microarray analysis of mouse brain tissue lacking Top2b highlighted misregulation of genes involved in neuronal functions such as axon guidance and synaptic function. Furthermore, Top2b was shown to bind and regulate neuronal genes in mouse embryonic stem cell-derived postmitotic neurons [13]. In that study, Top2b deletion led to degeneration of postmitotic neurons and was accompanied by downregulation of genes highly enriched in GO terms associated with neurogenesis. Moreover, neuronal degeneration observed in cells lacking Top2b was linked to upregulation of *Ngfr* p75 which was shown to be a direct target for Top2b through binding to its promoter and negatively regulating its transcription [13]. In addition, Top2b interacts with the chromatin remodeller Chd7 to regulate a set of long genes involved in neuronal function in mouse cerebellar granule neurons, and the Top2 catalytic inhibitor ICRF-193 led to downregulation of 203 of these genes. Lastly, Top2b has been reported to have a role in motor neurone (MN) connectivity and migration in mice, where terminal branching of MNs was remarkably defective in Top2b knockout mice. This was probably due to improper Hox/Pbx-dependent transcriptional activation [26].

In the developing immune system, TOP2B hypomorphs are the cause of Hoffman syndrome, characterised by a lack of mature B cells which in a mouse model system is associated with failure to activate transcription of the key B cell factor *Pax5* [15].

In addition to its role in neural and immune system development, TOP2B facilitates transcriptional activation in response to stimuli such as heat shock, serum induction [27], neuronal [28], and nuclear hormone receptor ligands [29, 30]. A theme emerging from these studies is a DNA strand break at or near the promoter of the activated genes that may be mediated by TOP2B. For example, retinoic acid (RA) treatment of MCF7 cells results in the appearance of a DNA break in the promoter of the RA-inducible retinoic acid β (*RARB*) gene [29]. In an independent study in NB4 cells, TOP2B was shown to associate with the retinoic acid receptor α (RARA) and to negatively modulate its transcriptional activity in acute promyelocytic leukaemia (APL) cell lines leading to RA resistance. TOP2B knockdown in RA-resistant clones released a transcription block and allowed RA-induced differentiation. TOP2B overexpression in RA-sensitive clones was able to induce RA resistance by reducing expression of RA target genes. In addition, chromatin immunoprecipitation (ChIP) analysis showed that TOP2B is specifically bound to the retinoic acid response element (RARE) region of the promoter of RARB gene which is one of the RA target genes [31].

SH-SY5Y is a neuroblastoma cell line that can be induced by RA to differentiate into adrenergic neuronal-like cells [32–35], making these cells a good model system to study the role of TOP2B in both RA-induced transcription and RA-induced differentiation of neuronal cells. The SH-SY5Y cell line is composed of a heterogeneous cell population, a characteristic feature of neuroblastoma cells. Although these subpopulations show different biochemical and phenotypic characteristics, they are not genetically distinct, as one subpopulation can switch into another [36, 37] characteristic of epigenetic changes. The majority of the SH-SY5Y population are N-type cells, characterised by small, rounded body shape with limited cytoplasm and short neurite processes. N-type cells grow as weakly attached cell aggregates and bear neuronal biochemical properties such as neurotransmission enzyme activity including tyrosine hydroxylase (TH) and dopamine-β hydroxylase (DBH) [36, 38, 39]. S-type cells, which typically constitute a small percentage of the cell population, are characterised by larger cell body size, expansive cytoplasm, flattened appearance, stronger attachment properties, epithelial-like morphology, and expression of the mesenchymal intermediate filament gene vimentin (*VIM*) [39, 40]. A third rare category, I-type cells, represents an intermediate state between N- and S-type. Recent transcriptome and ChIP-seq analyses support the conclusion that both primary tumours and neuroblastoma cell lines resemble either committed adrenergic cells or undifferentiated neural crest mesenchymal cells and that these states are epigenetically established via super-enhancer networks. N-type SH-SHSY have the expression profile of the former cell type, and S-type cells the latter [41–44].

Retinoic acid induces cell cycle exit and neuronal differentiation in SH-SY5Y cells. Differentiated cells have a rounded cell body with long, straight, less branched neurite outgrowths [34, 45] and exhibit upregulation of genes such as neurotrophic tyrosine kinase 2 (*NTRK2*) [46, 47] that are associated with neurogenesis as well as genes including *CRABP2* and *CYP26A1* and *RARB* (see Table S1) that are involved in retinoid intracellular transport and metabolism or signalling [48].

We have utilised the SH-SY5Y cell line model to study the effect of TOP2B on RA-induced transcription and neurone-like potential by generating a TOP2B knockout model and comparing knockout cells with wild-type cells in the presence and absence of ATRA for differentiation and whole-genome expression.

## Materials and Methods

### Cell Culture

SH-SY5Y clones were maintained in a 50:50 mixture of minimum essential medium (MEM) and F12 (Hams) medium (Gibco by Life Technologies, Invitrogen, UK) containing 10% v/v heat-inactivated foetal bovine serum (FBS) (Gibco by Life Technologies, Invitrogen, UK) and 1% v/v penicillin and streptomycin solution (10,000 units/ml penicillin, 10,000 mg/ml streptomycin, Gibco by Life Technologies, Invitrogen, UK). SH-SY5Y cells were maintained at 75–80% confluency and subcultured by trypsinisation with 0.05% Trypsin–EDTA (Gibco by Life Technologies, Invitrogen, UK) at 37 °C for 5 min.

### RT-PCR

Cells were grown in 96-well plates, and RNA samples used for RT-qPCR were prepared directly from fresh cell lysates using SingleShot Cell Lysis Kit (Bio-Rad) according to manufacturer’s instructions as previously described [49].

Gene expression analysis was performed by real-time one-step RT-PCR using SYBR Green I detection method using the primers shown in Table 1. For this purpose a QuantiNova™ SYBR Green RT-PCR Kit (QIAGEN, Cat. 208,154) was used according to the manufacturer’s instructions. Briefly, the total reaction volume was set to 20 μl per well in the PCR 96-well plate. In each reaction well, the following volumes were added: 10 μl (2 × SYBR Green RT-PCR), 300 nM primer mix, 0.2 μl (QuantiNova SYBR Green RT Mix which contains HotStaRT-Script reverse transcriptase), and 1–2 μl sample volume, and the volume was completed to 20 μl with RNase-free water. Contamination was monitored with two controls: (no reverse transcriptase) control to determine DNA contamination in the samples and (no template) control to determine other contaminations. The thermal cycler (CFX96 Touch™, Bio-Rad) was set to the following parameters: reverse transcription reaction (50 °C for 10 min), DNA polymerase activation and initial DNA denaturation (95 °C for 3 min) for 1 cycle, denaturation, and annealing and extension (95 °C for 15 s, 55 °C for 30 s, 72 °C for 30 s) for 40 cycles.

**Table 1:** RT-PCR oligonucleotides

Target	Primer	F/R	Sequence	Length (nt)	Amplicon size (bp)
*PP1A*	PP1A-1F	F	TCCTAAAGCATACGGGTCCTGGCAT	25	166
*PP1A*	PP1A-1R	R	CGCTCCATGGCCTCCACAATATTCA		
*TOP2B*	TOP2B_1F	F	ACCTGGGTGAACAATGCTGC	20	212
*TOP2B*	TOP2B_1R	R	ACCTCCCTGCAATTCATTCCT		
*CYP26A1*	CYP26A1-1F	F	GCAGCCACATCTCTGATCACT	21	109
*CYP26A1*	CYP26A1-1R	R	TGTTGTCTTGATTGCTCTTGC	21	
*RARB*	RARB-2F	F	GCAGAGCGTGTAATTACCTTGAA	23	145
*RARB*	RARB-2R	R	GTGAGATGCTAGGACTGTGCTCT	23	
*RET*	RET1F	F	GCTCCACTTCAACGTGTC	18	158
*RET*	RET1R	R	GCAGCTTGTACTGGACGTT	19	
*CRABP2*	CRABP1-F1	F	TCAAAGTGCTGGGGGTGAAT	20	168
*CRABP2*	CRABP1-R1	R	TCTGCTCCTCAAACTCCTCCC	21	
*BCL2*	BCL2-3F	F	GATTGTGGCCTTCTTTGAG	19	232
*BCL2*	BCL2-3FR	R	CAAACTGAGCAGAGTCTTC	19	
*NTRK2*	NTRK2-1 F	F	CAATTGTGGTTTGCCATCTG	20	238
*NTRK2*	NTRK2-1R	R	TGCAAAATGCACAGTGAGGT	20	
*VIM*	VIM-F	F	GACAGGATGTTGACAATGCG	20	222
*VIM*	VIM-R	R	GTTCCTGAATCTGAGCCTGC	20	

### Generation of SH-SY5Y TOP2B.^−/−^ Clones Using CRISPR-Cas9

TOP2B null cell lines were generated by targeting TOP2B exon 1 with the guide RNAs shown in Table 2 cloned into pSpCas9 (BB)-2A-GFP (PX458) (Addgene plasmid # 48,138). The plasmid was transfected into SH-SY5Y cells using Nucleofection with Amaxa Nucleofector II system and the Cell Line Nucleofector® Kit V (Lonza, UK, cat. VCA-1003) according to the manufacturer’s instructions. Cells were selected and sorted based on GFP expression as described previously [49]. GFP-positive cells were incubated at 37 °C for 2–3 weeks until colonies formed. A control cell line was generated by transfecting cells with PX458 containing no guide RNA and selecting as above. Screening for TOP2B knockout clones was performed using PCR genotyping and immunofluorescence and confirmed by DNA sequencing and Western blotting.

**Table 2:** Guide RNA sequences

gRNA	Sequence 5′ to 3′	Strand	TOP2B^−/−^ clone
5	GCGGCAACGGGGCACTGACC	-	BKO70
6	CGCGCCGCAGCCACCCGACT	+	BKO98
7	AAGTCGGGTGGCTGCGGCGC	-	BKO129

### RNA-seq

Total RNA was prepared from both wild-type and TOP2B null BKO98 cells treated for 24 h with either 1 µM ATRA in ethanol or an equivalent volume of ethanol alone. Four biological replicate RNA samples were prepared for each of the four conditions. RNA extraction, quantification, and quality checking were as previously described [49]. Library preparation employed TruSeq-stranded mRNA kit (Illumina) which selects only poly-A containing mRNA molecules using poly-T oligo attached magnetic beads using the High Sample (HS) Protocol of (TruSeq® Stranded mRNA Sample Preparation Guide, Illumina) was followed. The input RNA for library preparation was 1500 ng per sample. Sequencing was performed using the Illumina NextSeq 500 sequencing system. A high output flow cell was used, and 75 bp single-end (SE) sequencing was performed.

Transcript-level quantification was performed using Salmon [50]. Subsequent analysis steps employed R software (version 3.4). The principal component analysis (PCA) and differential gene expression were performed using the R Bioconductor package DESeq2 [51]. Differential gene expression comparisons were performed pairwise between each of the four conditions. Gene annotation was added using the annotables package with GENCODE release 29 (GRCh38.p12) as reference annotation. Volcano plots and heat map were prepared using R 4.0.3.

### Neurite Outgrowth Assays

Initial number of seeded cells was optimised in such a way that both ATRA-treated and untreated cells had a comparable confluency by the end of time course of differentiation (7 days). Cells were treated with either (10 μM) ATRA or ethanol (0.01% V/V) for 7 consecutive days, and media was replaced every 2 days. Cells were then imaged by phase-contrast microscopy using a TiE fluorescence wide-field inverted microscope (Nikon). At least six fields were captured for each sample, images were analysed, and neurite lengths were measured using ImageJ.

### Cell Migration Assays

Cell migration propensity was determined using wound healing assays in starvation medium consisting of reduced serum normal growth medium (0.1% FBS). Cells were plated into 35-mm dishes. Almost confluent monolayers of cells were scratched using a micropipette tip making five parallel scratches per dish. Ten images were recorded per plate immediately after scratching and after 6 and 24 h. Gap width was measured using ImageJ with the plugin *Wound_healing_size_tool* [52].

## Results

### Retinoid Response and TOP2

**Fig. 1:**
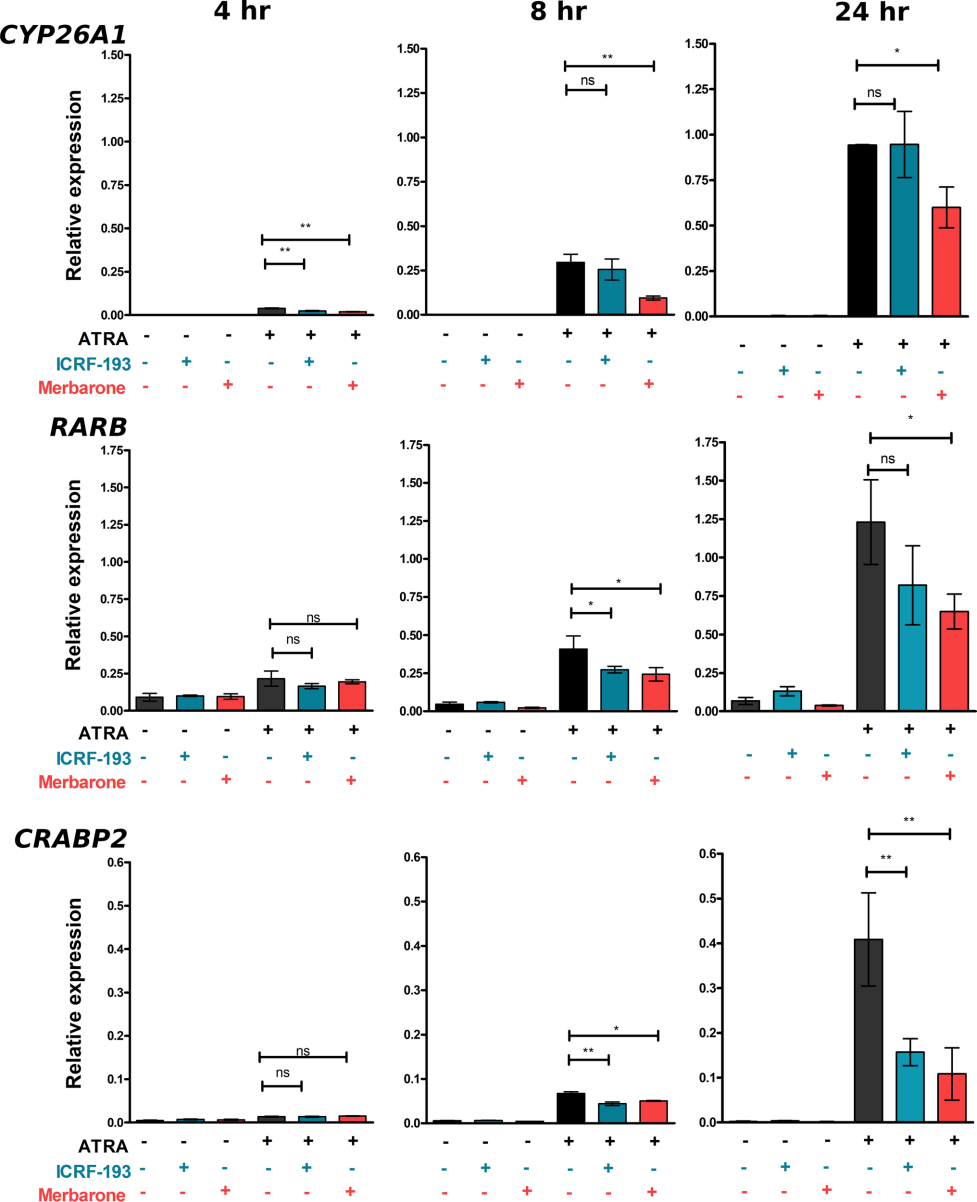
TOP2 catalytic inhibitors suppress ATRA-induced expression of *CYP26A1*, *RARB*, and *CRABP2.* SH-SY5Y cells were incubated with ATRA in the presence or absence of one of the TOP2 catalytic inhibitors, merbarone or ICRF-193 for the times indicated. Expression data was calculated relative to the control housekeeping gene PP1A. Significance testing was performed using 1-way ANOVA with Dunnett’s post-hoc test. Error bars are SDs

The retinoid response in the SH-SY5Y cells was first confirmed by assessing the induction of three genes that have been reported to be regulated by retinoic acid. *CYP26A1*, *CRABP2*, and *RARB* (all gene symbols and corresponding gene/protein names are listed in Table S1) were previously demonstrated to be rapidly induced in SH-SY5Y and other neuroblastoma cell lines treated with retinoids [47, 48, 53]. *CYP26A1*, *CRABP2*, and *RARB* RNAs were robustly induced after treatment with 1 µM *all-trans* retinoic acid (Tretinoin, ATRA) for 24 h (Fig. S1A). Notably, *TOP2B* expression was not affected by ATRA treatment over this period (Fig. S1B). We found that, as expected, TOP2B protein levels were also maintained at a high level over a longer period of ATRA treatment (5 days, see Fig. S2 A&B), while in most cells, TOP2A was dramatically downregulated (Fig. S2C and S2D). To test the requirement for TOP2 activity in ATRA-induced gene expression, SH-SY5Y cells were treated with ATRA as before, but also with either the TOP2 catalytic inhibitors ICRF-193 or merbarone or solvent control [54, 55]. Cells were collected for RT-PCR at 4, 8, and 24 h after ATRA addition. Increased *CYP26A1*, *RARB*, and *CRABP2* mRNA was measurable by 4 h, and this was more pronounced for each gene after 8 and 24 h. At these later time points, merbarone significantly supressed ATRA-induced accumulation of RNA of all three genes. ICRF-193 treatment had a small effect on ATRA-induced *RARB* expression and a more robust effect on ATRA-induced expression of *CRABP2* (Fig. 1). Notably though, not all ATRA-induced gene expression was inhibited by these compounds. For example, the RET receptor tyrosine kinase (*RET*) which is robustly induced by retinoids, and which plays a key role in transcriptional events related to RA-induced differentiation, was unaffected by merbarone or ICRF-193 (Fig. S3A). In addition, expression of TOP2B itself was not significantly affected by either ATRA or merbarone (Fig. S3B). These results support a role for TOP2 activity in the induction of some, but not all genes that respond to ATRA in SH-SY5Y cells. However, these inhibitors do not allow us to determine the relative contribution of TOP2A and TOP2B to ATRA-induced gene expression changes. To address this question and to determine the role of TOP2B in establishing and maintaining gene expression patterns in SH-SY5Y cells in general, we constructed a series of *TOP2B* null cell lines employing CRISPR-*Cas9* targeting exon one of *TOP2B*.

### Generation and Characterisation of TOP2B CRISPR Knockouts

**Fig. 2:**
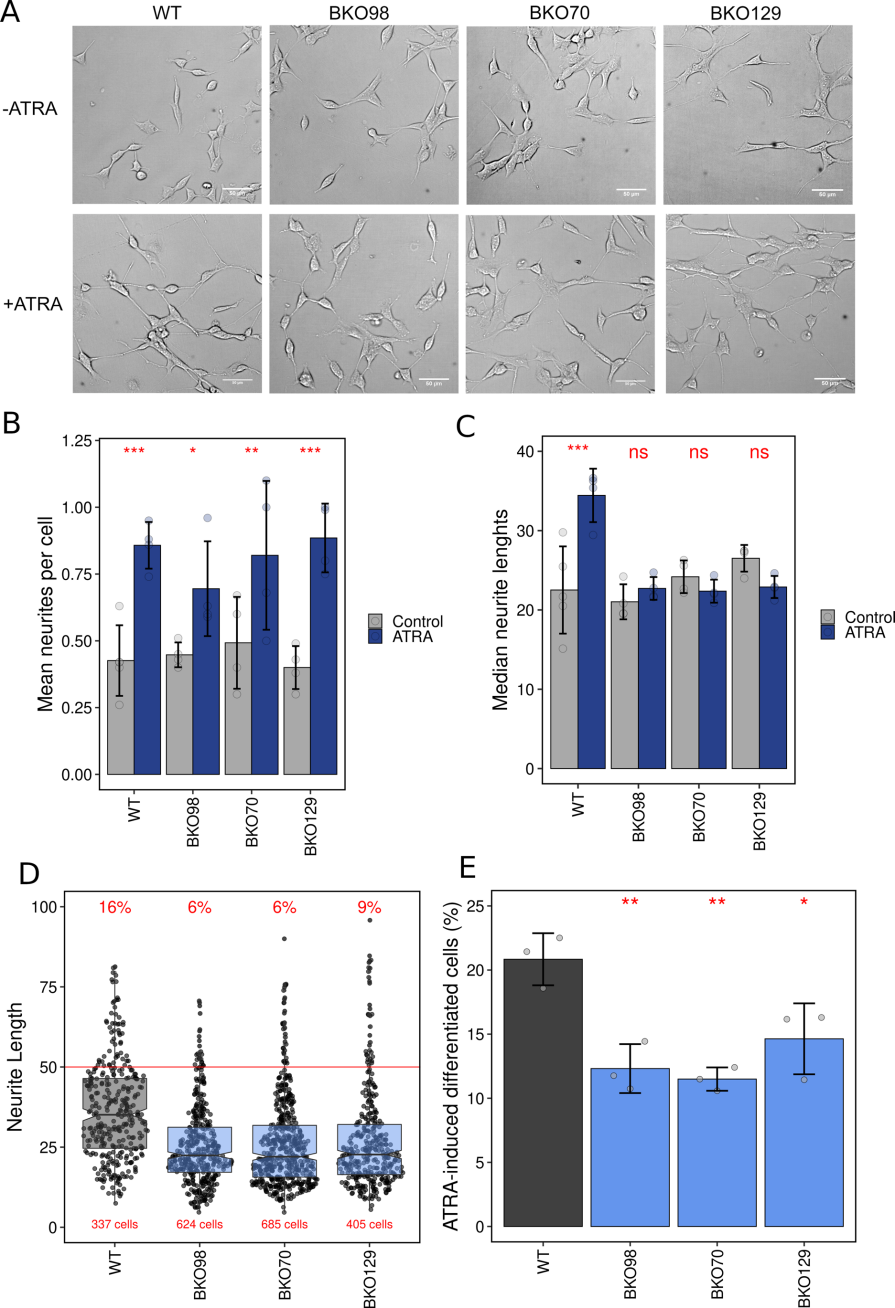
Morphological appearance and differentiation of wild-type and TOP2B null SH-SY5Y clones. (**A**) Phase-contrast images of WT and TOP2B null cell lines BKO98, BKO70, and BKO129 treated with either vehicle (EtOH, -ATRA) or 10 µM ATRA (+ ATRA) for 7 days. (**B**) Neurite outgrowths per cell were counted for control and ATRA-treated cells. The mean number of neurites counted per cell are plotted. (**C**) Neurite lengths were measured, and the median length calculated for each replicate. Means of medians are plotted. (**D**) Lengths of neurite outgrowths in ATRA-treated cells (data from all replicates combined) are plotted as a scattergram. The percentage of neurites that were longer than 50 µm and the total number of cells is indicated in red. (**E**) The percentage of cells exhibiting one or more neurites longer than 50 µm under control or ATRA-treated conditions was determined. The mean difference between control and ATRA-treated cells is plotted. Error bars represent 1 standard deviation. For (**B**), (**C**), and **(E**), the small circles represent the values from individual replicates. For (**B**) and (**C**), significance testing was performed by 2-way ANOVA with Bonferroni’s post-hoc test, and for (**E**), significance was tested using 1-way ANOVA with Dunnett’s post-hoc test (*P ≤ 0.05, **P ≤ 0.01, ***P ≤ 0.001)

Several TOP2B null SH-SY5Y cell lines were generated using three different guide RNAs. One line derived from each guide RNA was selected for further study. These three lines are henceforth referred to as BKO70, BKO98, and BKO129 (see Fig. S4). Each of these *TOP2B* null cell lines proliferated robustly in culture and had a similar appearance to wild-type cells (see Fig. 2A) with similar numbers and length distribution of neurites under normal growth conditions (Fig. 2B and C). Retinoic acid treatment induces neurite outgrowth in neuroblastoma cells such as SH-SY5Y, and this can be used as a measure of differentiation in these cells [34]. To determine the effect of loss of TOP2B on ATRA-induced differentiation, neurite outgrowths induced by ATRA were measured in wild-type and knockout cells. SH-SY5Y cells and BKO70, BKO98, and BKO129 cells were treated with either (10 μM) ATRA or an equal volume of solvent (ethanol, 0.01% V/V) for 7 consecutive days, and media was replaced every 2 days to keep ATRA concentration consistent over the differentiation period. Cells were then imaged by phase-contrast microscopy, and neurite lengths and numbers were determined. ATRA treatment resulted in an increase in the number of neurites counted per cell for both WT and TOP2B null cell lines (Fig. 2B), but produced a significant increase in median neurite length only in WT cells (Fig. 2C and D). Neurite outgrowths of more than 50 μM have been used as a marker of ATRA-induced differentiation in SH-SY5Y cells [34, 56]. The TOP2B knockout clones showed a significant reduction in the ratio of cells bearing neurite outgrowths of > 50 μM (Fig. 2D and E). This is consistent with previous findings using the TOP2 inhibitor ICRF-193 that implicate Top2b in neurite outgrowth in cultures mouse neurones [18].

### TOP2B Knockout Clones Exhibit Altered Transcriptional Response to ATRA

**Fig. 3:**
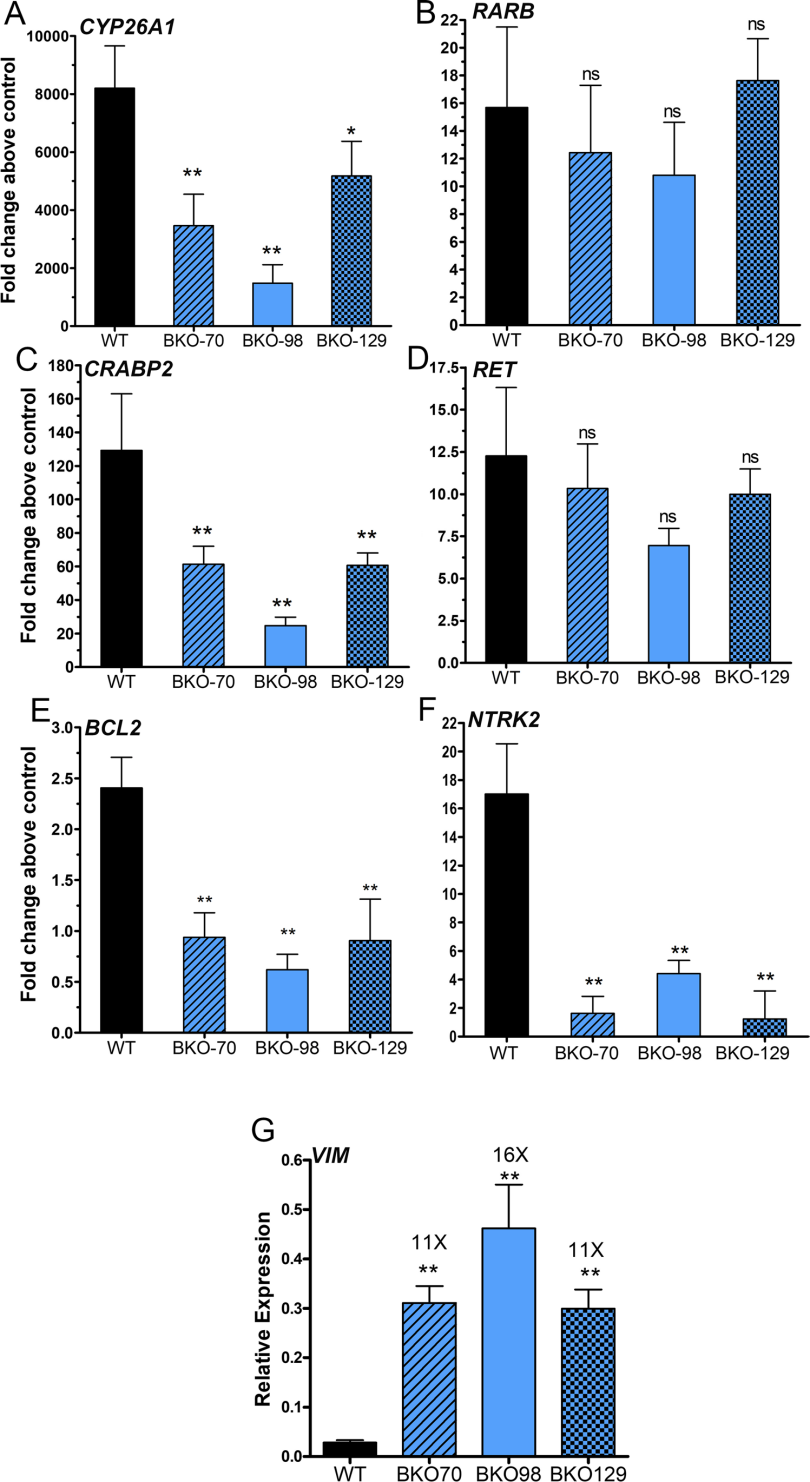
ATRA-induced gene expression in WT SH-SY5Y cells and TOP2B null clones. (**A**–**F**) Relative expression was determined by RT-qPCR using *PP1A* gene as a normalisation reference. Cells were treated with or without 1 μM ATRA, and the ΔΔCt method was used to calculate the fold change in induction above control for each clone. Data presented here is the mean of at least three independent experiments ± SD. (**G**) Relative expression in vimentin (*VIM*) in WT SH-SY5Y and TOP2B null clones. Significance tests were performed using 1-way ANOVA with Dunnett’s post-hoc test

To examine the requirement for TOP2B in retinoid-induced gene expression, we compared the expression of *CYP26A1*, *CRABP2*, *RARB*, and *RET* by quantitative RT-PCR in wild-type versus the three TOP2B null cell lines after exposure of cells to ATRA for 24 h. ATRA-induced *CYP26A1* and *CRABP2* expression was significantly reduced in all three null cell lines. However, ATRA induction of *RARB* and *RET* was not significantly affected (Fig. 3A–D). Next, we tested the expression of *NTRK2* (neurotrophic receptor tyrosine kinase 2, TrkB) and *BCL2* (B cell CLL/lymphoma 2); both genes linked to neural differentiation whose expression is increased in RA-treated neuroblastoma cells [34, 46, 57–60]. As expected, both *BCL2* and *NTRK2* were significantly induced by ATRA in wild-type cells (approximately 2.5-fold and 17-fold, respectively). However, in each of the three TOP2B null cell lines, ATRA failed to induce *BCL2* above control levels, and ATRA-induced expression of *NTRK2 *was significantly reduced (Fig. 3E and F).

### TOP2B KO Cells Express Vimentin

Since induction of neural differentiation markers *BCL2* and *NTRK2* was suppressed, we compared the expression of *VIM*, a characteristic marker of S-type neuroblastoma cells [34, 42], in untreated wild-type and TOP2B null SH-SY5Y cells. *VIM* expression was at least ten times higher in the TOP2B null than in the wild-type cells (Fig. 3G), even though these cells did not have the flat, adherent phenotype [34] of S-type SH-SY5Y cells (Fig. 2A).

### Differential Expression Analysis

**Fig. 4:**
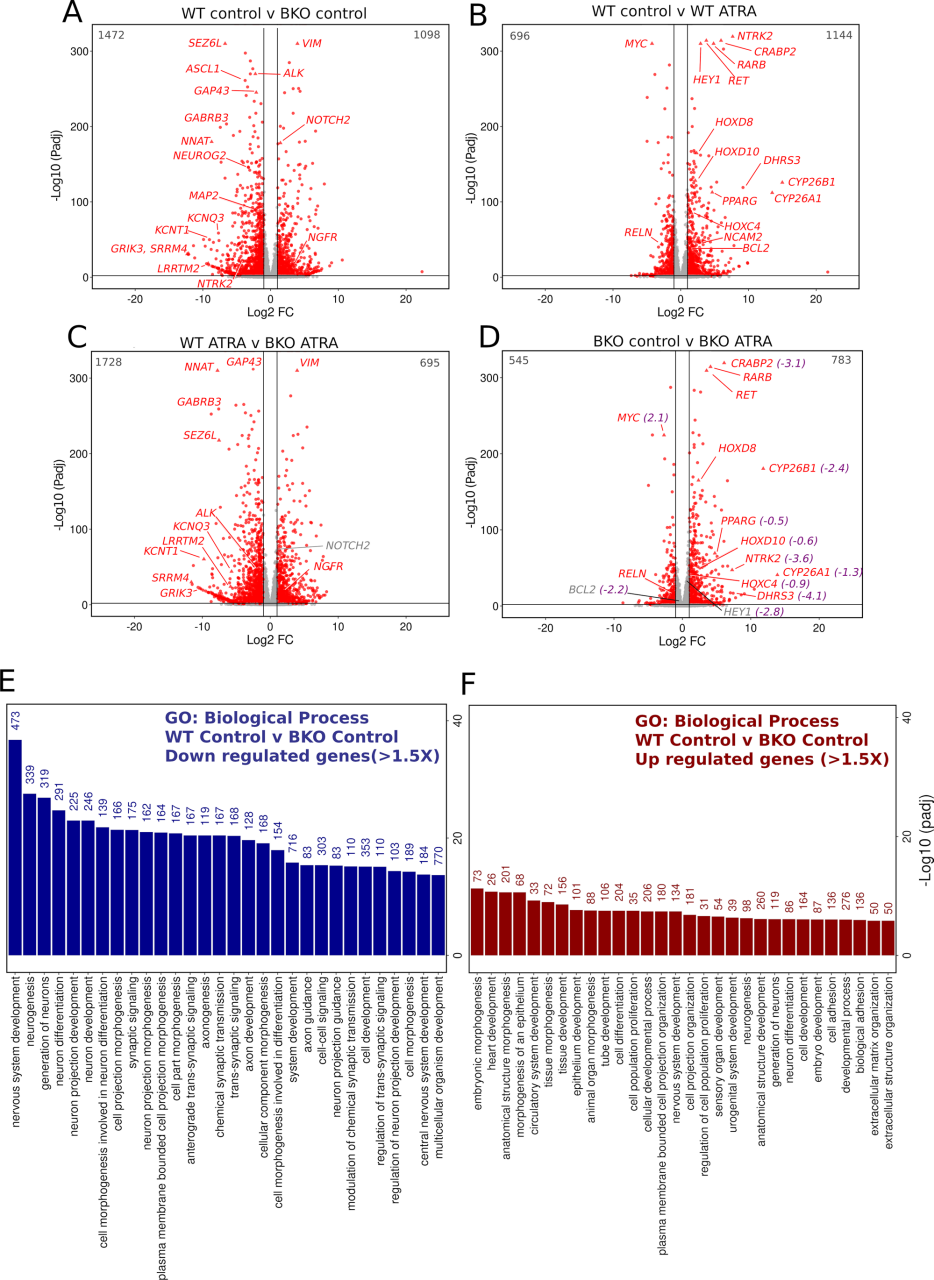
Differentially expressed genes: WT versus TOP2B null SH-SY5Y cells under control and ATRA-treated conditions. (**A**–**D**) Volcano plots showing differential gene expression in 4 different comparisons in SH-SY5Y cells or the TOP2B null clone BKO98. (**A**) WT cells versus BKO98 cells, vehicle control. (**B**) WT cells, vehicle control versus WT cells ATRA treatment. (**C**). WT cells versus BKO98 cells after ATRA treatment. (**D**) BKO98 cells, vehicle control versus BKO98 cells ATRA treatment. Transcripts that were significantly (Padj < 0.05) either up- or downregulated by more than 1 log2 fold are labelled red. Specific genes mentioned in the text are labelled. In (**D**) Log2FC (WT control versus BKO control) values are included in purple if the respective genes were over- or under-expressed in untreated BKO cells by more than 1.5 × . (**E** and **F**) Biological process terms associated with genes down- (**E**) or (**F**) upregulated > 1.5 × in TOP2B null (BKO98) versus WT SH-SY5Y cells. Analysis was performed using g:GOSt (https://biit.cs.ut.ee/gprofiler/gost), non-ordered, significance threshold Benjamini–Hochberg FDR < 0.05) [67]. Only the top 30 terms by significance value are shown. Intersect size is shown above each bar

To fully explore both differences in gene expression profiles between WT and TOP2B null SH-SY5Y cells, and differences in the transcriptional response to ATRA, we carried out RNA-seq analysis using four biological replicates that were prepared from both SH-SY5Y and TOP2B-null BKO98 cells either treated with ATRA or vehicle for 24 h. The four biological replicas for each condition clustered tightly together, and the effect of genotype and ATRA treatment clearly separated in principle component analysis (Fig. S5A). Comparison of gene expression profiles observed between the two cell lines and two conditions is illustrated in the heatmaps in Fig. S5B and S5C. Differential gene expression (DEG) analysis was carried out with four comparisons: Untreated WT SH-SY5Y cells versus untreated BKO98 cells (WT control vs BKO control), untreated WT SH-SY5Y cells versus ATRA treated WT SH-SY5Y cells (WT control vs WT ATRA), untreated versus ATRA treated BKO98 cells (BKO control vs BKO ATRA), and wild-type ATRA-treated cells versus ATRA-treated BKO98 cells (WT ATRA vs BKO ATRA). The WT control vs BKO control analysis revealed many genes that were significantly (Padj < 0.05) either up- or downregulated in the TOP2B null BKO98 cells compared to WT cells. One thousand four hundred seventy-two transcripts were downregulated by two-fold or greater in the TOP2B null cell line, while 1098 were upregulated (Fig. 4A and Online resource 1). Genes with known functions in neural differentiation and function were amongst the most highly downregulated genes; examples are highlighted in Fig. 4A and Tables S1S2S3. Upregulated genes included *VIM* (see above), the cell fate signalling gene *NOTCH2*, and nerve growth factor receptor (p75) gene *NGFR* (Fig. 4A, Table S1). Many of the same genes were also differentially regulated between WT and TOP2B null cells under ATRA treatment conditions (i.e. WT ATRA versus BKO98 ATRA) (Fig. 4C). ATRA treatment resulted in a larger number of upregulated than downregulated genes in both WT and BKO cells (Fig. 4C and D, Online resource 1), although the number of genes significantly changed by more than two-fold was greater in the WT cells.

### TOP2B Null SH-SY5Y Cells Have Reduced Adrenergic and Increased Mesenchymal Transcriptome Properties

**Fig. 5:**
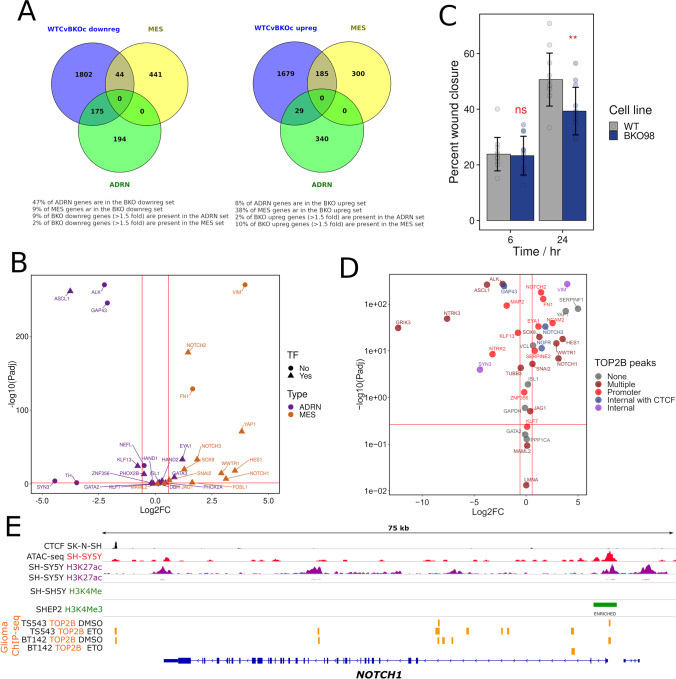
The profile of up- and downregulated genes in BKO98 cells suggests a transition towards a more undifferentiated mesenchymal state in TOP2B null cells. (**A**) Intersections between genes downregulated (left) or upregulated (right) in BKO98 cells (greater than 1.5-fold, Padj < 0.05) and genes characteristic of committed adrenergic neurone-like (ADRN) or undifferentiated mesenchymal cell-like (MES) neuroblastoma cells [42]. Intersections are plotted as Venn diagrams. (**B**) Volcano plot of WT SH-SY5Y versus BKO98 Log2 fold change values, showing key genes that characterise the ADRN (purple) or MES (orange) states. (**C**) Cell migration of WT and BKO98 cells was compared using a wound healing assay. Nearly confluent monolayers of cells were scratched with a pipette tip and percentage “would closure” was measured from phase-contrast images after 6 or 24 h in starvation medium. Data are mean values + / − standard deviation. (**D**) Plot illustrating the relationship between the presence of TOP2B binding peaks and gene expression changes between WT and BKO cells. Genes are categorised according to the evidence for TOP2B binding from inspection of a published glioma ChIP-seq data set [62] according to the following scheme: none, no peaks observed in the promoter region or gene body; multiple, multiple peaks present; promoter, peak or peaks present in the promoter region; internal with CTCF, TOP2B peak or peaks at a gene-internal position coincident with CTCF binding; and internal, peak or peaks within the gene, not associated with CTCF. (**E**) Profile of epigenetic features, including glioma TOP2B binding activity [62] for *NOTCH1*, profiles for other relevant genes are shown in Fig. S8. Data were plotted using the Integrated Genome Viewer (IGV, Broad Institute) [75] using the following data sources (GEO accession and citation): SK-N-SH CTCF ChIP-seq, GSM2038347; SH-SY5Y ATAC-seq, GSM2700775 [76]; SH-SY5Y H3K27ac, GSM3676080 [77]; GSM2038349; SH-SY5Y H3K4Me3, GSM2419933 [41, 42]; SH-EP H3K4Me3, GSM2419932 [41, 42]; and TOP2B ChIP-seq data from TS543 and BT142 glioma cell lines in the absence (DMSO) or presence of etoposide (ETO), GSM3904399, GSM3904400, GSM3904401, and GSM3904402 [62]

SH-SY5Y cells exist as different interchangeable types, the predominant N-type with adrenergic neuronal properties, and the more epithelial mesenchymal S-type, as well as an intermediate I-type [32–35]. N- and S-type cells resemble and presumably reflect committed adrenergic and undifferentiated NC mesenchymal neuroblastoma cell types, respectively [41–44]. We observed that neuronal markers including *NTRK2*, *SEZ6L*, *MAP2*, *GAP43*, and *NEFM*, and key neuronal commitment genes *ASCL1* and *NEUROG2* were downregulated in TOP2B null SH-SY5Y cells (Fig. 4A, Tables S1 and S3, Online resource 1), while as described above, the mesenchymal intermediate filament vimentin gene (*VIM*) was dramatically upregulated. This, together with reduced ATRA-stimulated neurite outgrowth in the BKO cells (Fig. 2A), suggested that the TOP2B null genotype may result in a general loss of adrenergic neuron-like transcriptional signatures and gain of undifferentiated mesenchymal properties. This was supported by functional gene enrichment analysis (see Fig. 4E and F and Fig. S6). Genes downregulated in BKO cells are enriched in GO:Biological process (BP) terms including nervous system development, neurogenesis, neurone projection morphogenesis, and axon guidance (which include the *ASCL1*, *NEUROG2*, *ALK*, *GAP43*, *SEZ6*, *NEFL, NTRK2*, *TH*, *MAP2, PHOX2B*, and *HAND2* genes). Similarly, nine of the ten KEGG pathways significantly enriched for downregulated genes are related to neuronal function or neural development, the most significant of which is axonal guidance (Table S4, Fig. S7) [32–35]. Genes upregulated in the TOP2B null cells are enriched in largely non-neuronal-related GO:BP terms including Embryonic Morphogenesis, Morphogenesis of an Epithelium (Figs. 4F and S6), terms which include *NOTCH1, NOTCH2*, *YAP1*, *HES1*, and *SOX9*). Recent transcriptomic and epigenetic studies have highlighted super-enhancer associated transcription networks underlying the adrenergic neurone like (ADRN) and undifferentiated mesenchymal cell (MES) states of neuroblastoma cell lines [41–44]. Analysis of a range of independent neuroblastoma-derived cell lines generated a high confidence list of differentially expressed genes characteristic of these two states [42]. Forty seven percent of genes identified in the ADRN transcription profile in that study [42] intersect with genes downregulated in TOP2B null SH-SY5Y cells, while this was true of only 9% of MES genes. Conversely, 38% of genes identified in the MES transcription profile intersect with genes upregulated in TOP2B null SH-SY5Y cells, while only 8% of ADRN genes were present in the upregulated set (Fig. 5A and B). Thus, the transcriptional changes observed in TOP2B null SH-SY5Y cells resemble a transition towards a more MES-like state, consistent with increased expression of genes such as *VIM.* The directionality of the transcription changes is illustrated in Fig. 5B. Several key MES-associated genes including the transcription factors *NOTCH1-3* and *HES1* are strongly upregulated, while the ADRN-associated transcription factor *ASCL1* is strongly downregulated. N-type WT SH-SY5Y cells fall within ADRN transcriptional profile [42] and exhibit low migratory capacity, while the MES profile is associated with greater cell migration [41, 61], and SH-SY5Y cells have been shown to adopt MES-type characteristics following exogenous expression of NOTCH intracellular domains [41, 61]. Since BKO98 cells express elevated levels of *NOTCH* RNA, we compared the migration capacity of WT SH-SY5Y and BKO98 cells. Unexpectedly, the TOP2B null cells exhibited slightly reduced migration in wound healing assays (Fig. 5C). This suggests that although the transcriptional profile of BKO98 cells supports a move towards a more MES-like phenotype, this transition is partial and not fully reflected in cellular properties.

The expression of nearly 2500 genes is altered in the BKO98 cell line. It is likely that some of these gene expression changes are a direct consequence of loss of TOP2B activity in gene regulatory regions, whereas others may result from indirect effects or a less localised effect on DNA supercoiling. To examine TOP2B binding in the gene regulatory regions of differentially expressed genes such as *NOTCH1*, we used an existing TOP2B ChIP-seq data set derived from glioma cell lines TS543 and BT142 [62] along with publicly available data for CTCF, open chromatin (ATAC-seq), active promoters (H3K4Me3), and promoter/enhancer regions (H3K27Ac), all derived from SH-SY5Y cells or clonally related lines (SK-N-SH or SHEP). Although the TOP2B ChIP-seq data was derived from a different neural cell type (glioma rather than neuroblastoma), inspection of the accompanying expression data revealed that most genes of interest from our RNA-seq analysis were also expressed in the glioma cells. Examination of the promoter region and gene body revealed peaks of TOP2 binding in the glioma data for most differentially expressed genes examined, whether they were up- or downregulated (Fig. 5D). This included *NOTCH1*, *NOTCH2*, and *NOTCH3* (Fig. 5D and E*,* Fig. S8). Thus, a direct requirement for TOP2B for normal expression of these genes is possible. However, some differentially expressed genes, including *YAP1* and *SERPINF1*, are not associated with TOP2B peaks, implying that for at least some genes, differential expression is an indirect effect.

### Transcriptional Response to ATRA Treatment in WT and BKO98 Cells

**Fig. 6:**
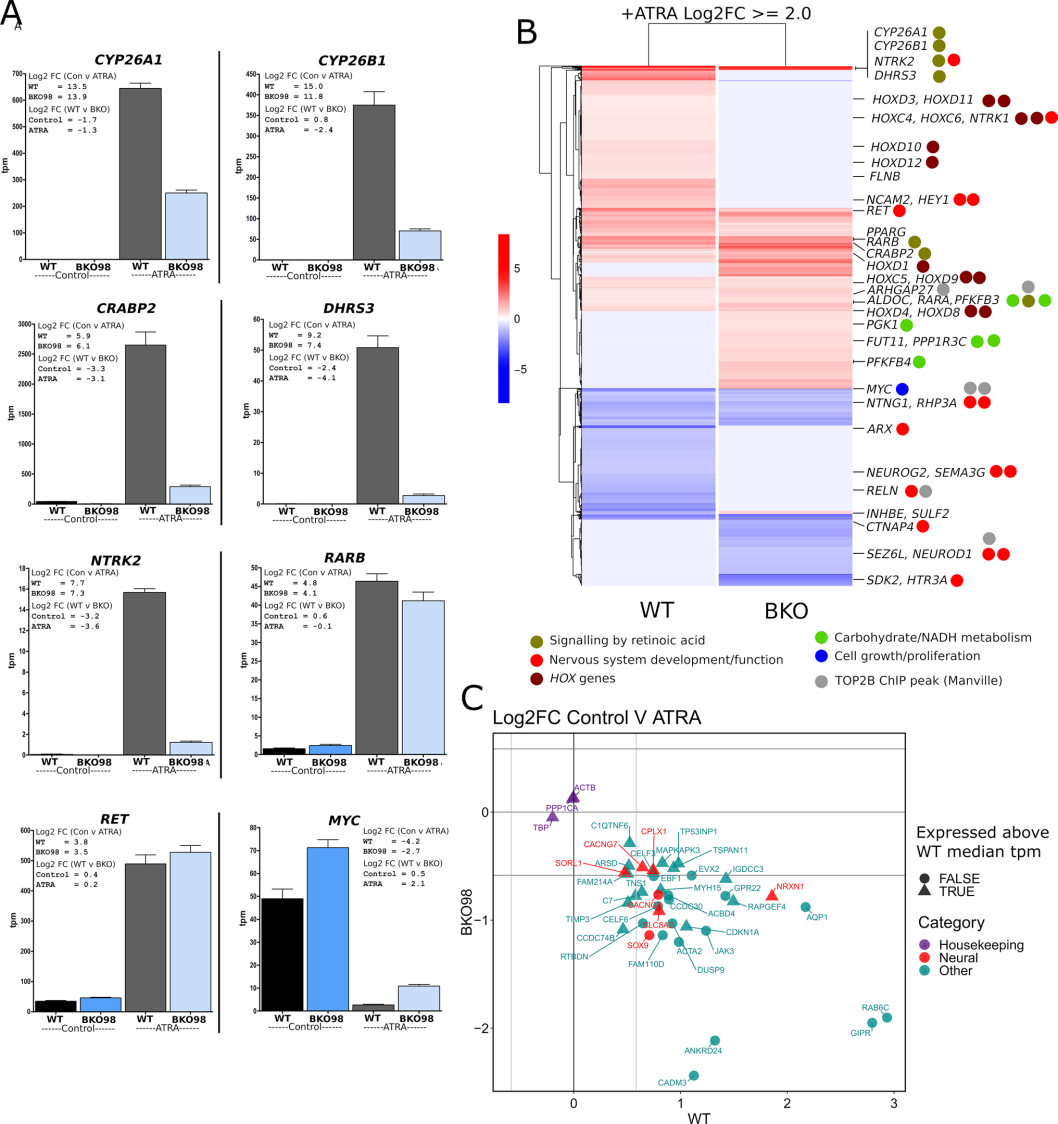
Differential expression of ATRA modulated genes in WT and TOP2B null (BKO98) SH-SY5Y cells lines. RNA-seq expression data is plotted to show relative expression levels (tpm, transcripts per kilobase million) for each gene in the four conditions, WT and BKO98, control, or ATRA treated. Log_2_ fold change (Log2FC) values are shown in insets. (**B**) Heatmap contrasting the transcriptional response to ATRA of WT SH-SY5Y and BKO98 cells. Significant genes are named and colour coded according to functional class (TOP2B ChIP-seq data from [68]). (**C**) Log2FC plot for genes whose expression is induced by ATRA in WT cells but reduced by ATRA in BKO98 cells highlighting in red genes that contribute to neural development or function Biological Process terms. For comparison, three housekeeping genes are also included. Genes are also classified according to their relative expression in WT untreated cells (above or below median tpm value)

As expected, ATRA treatment resulted in robust upregulation of *CYP26A1*, *CRABP2*, and a group of additional genes involved in retinoid metabolism, retinoid transport, and signalling in WT SH-SY5Y cells (Fig. 4B, Fig. 6A). This group of genes is listed in Table S2 and includes *CYP26B1*, *DHRS3*, and *RARB*. Some, but not all these genes are associated with well-defined retinoic acid response elements. The relative expression level (tpm value) of *CYP26A1*, *CYP26B1*, *CRABP2*, and *DHS3R* was considerably lower in BKO98 cells than in WT cells after ATRA treatment (Fig. 6A), implicating TOP2B in the induction of these genes by retinoic acid.

ATRA treatment also changed the expression of several neuronal differentiation markers, as has been previously reported [34, 35, 57, 63, 64]. Expression of *NTRK2*, *NTRK1, BCL2,* and *NCAM2* was significantly induced in WT cells (Table S3). *NTRK2* was expressed at a much lower level (> 10 ×) in BKO98 both before and after ATRA, but, in the RNA-seq analysis, had a similar ATRA-induced fold change in expression in both cell lines (Fig. 4B and D, Fig. 6A and B, Tables S1 and S3), while *NTRK1, BCL2* and *NCAM2* were not significantly induced in ATRA-treated BKO98 cells (Table S3). Similarly, several *HOX* genes including *HOXD11* and *HOXD12* which are known to be ATRA responsive in neuroblastoma cells, and to be able to induce growth arrest and differentiation in these cells [65], were significantly induced in WT but to a lesser degree or were not induced in BKO98 cells (Table S3, Fig. 6B). The *RET* tyrosine kinase was efficiently induced in both WT and TOP2B null cells (Fig. 6A and B), in agreement with TOP2 inhibitor studies and RT-PCR analysis of WT and TOP2B null cells (Fig. S3A, Fig. 3D, Fig. 4B and D, Fig. 6A).

In WT cells, ATRA treatment led to a greater than 18-fold downregulation of *MYC*, a cell cycle release gene that is well known for activating cyclins and cyclin-dependent kinases (CDKs). This was accompanied by a corresponding upregulation of the negative cell cycle regulator p21 (*CDKN1A*) [66]. *MYC* downregulation by ATRA was significantly more pronounced in the wild-type than the knockout cells (18-fold versus sixfold, Fig. 4B and D, Fig. 6A and B). This was accompanied by upregulation of *CDKN1A* in wild-type cells only (see Table S3).

ATRA treatment for 24 h did not cause significant change in TOP2A expression in either the wild-type or the knockout cells nor in TOP2B expression in the wild type, suggesting no direct effect of ATRA on either TOP2 isoform. By comparison, longer treatment with ATRA (5 days) caused a robust downregulation of TOP2A, but not TOP2B (see Fig. S2A). We also noticed that for a small number of genes (38), ATRA treatment resulted in upregulation in WT cells, but significant downregulation in BKO98 (Fig. 6C). Most of the expression changes for these genes in both WT and BKO98 cells are relatively small (Log2FC < 2) compared to a gene such as *NTRK2* which exhibits a Log2FC of over 7 in both cell lines. However, these data do illustrate that TOP2B can be necessary for the direction as well as magnitude of ATRA-induced gene expression changes.

**Fig. 7:**
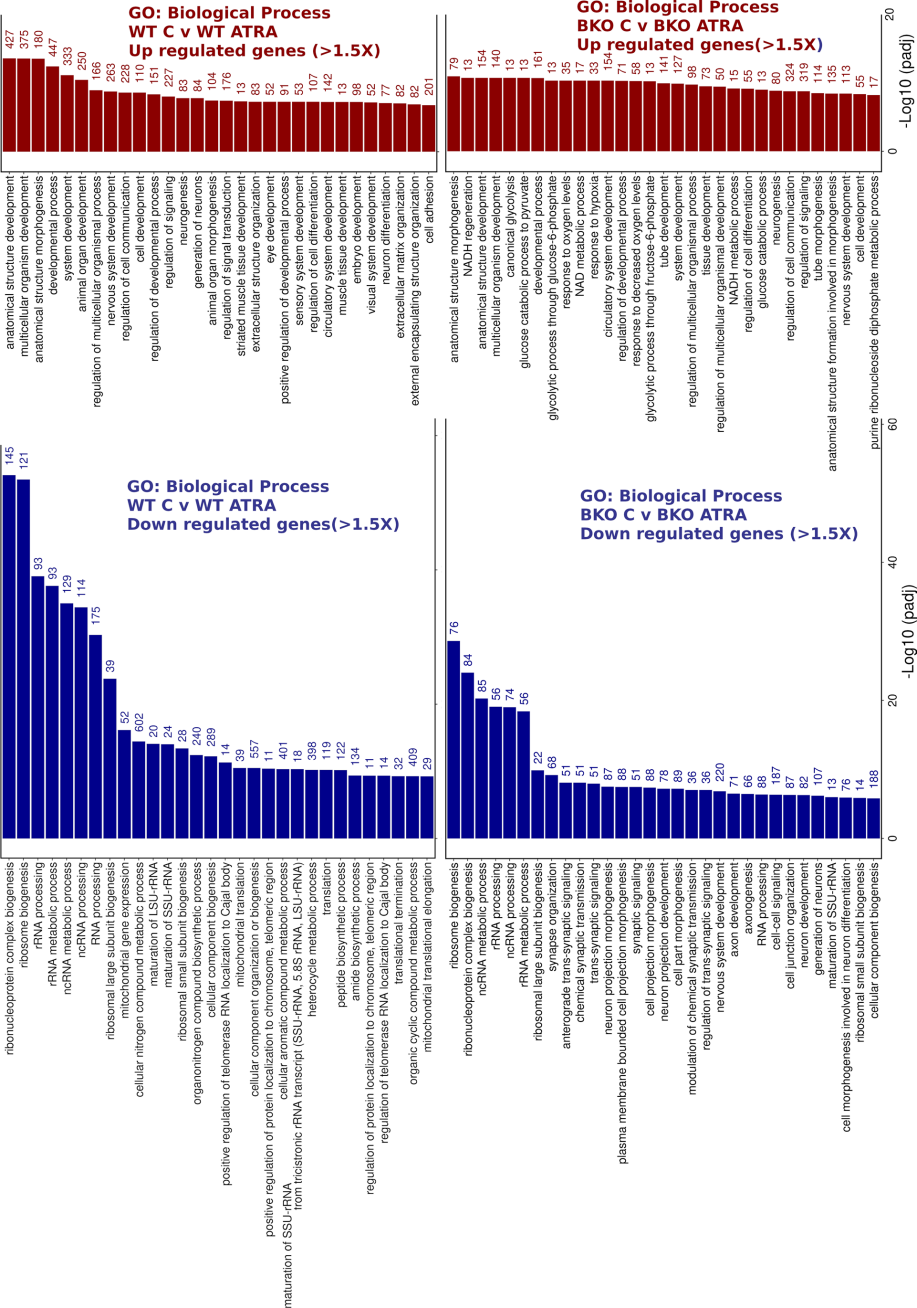
GO:Biological process terms associated with genes upregulated or downregulated > 1.5 × by ATRA treatment (24 h). Analysis was performed using g:GOSt (https://biit.cs.ut.ee/gprofiler/gost, non-ordered, significance threshold Benjamini–Hochberg FDR < 0.05) [67]. Only the top 30 terms by significance value are shown. Intersect size is shown to the right of each bar

Beyond individual genes, functional enrichment analysis [67] revealed clear differences in the GO: biological processes (BP) affected by ATRA treatment in WT versus TOP2B null cells. While for both genotypes, several morphological and development-related terms were amongst the 30 most statistically significant BP terms present for the upregulated genes, these terms contained fewer intersecting genes for the TOP2B null cells (Fig. 7). For example, “anatomical structure development”, “nervous system development”, and “neurogenesis” contained 472 versus 72, 263 versus 113, and 227 versus 80 intersections, respectively. This effect is borne out by clustering analysis, which also highlights glucose and NADH metabolic processes as significant BP terms only for the TOP2B null cells (Fig. S9). ATRA-downregulated BP terms with the greatest statistical significance that are shared between WT and TOP2B null cells are mostly involved in rRNA processing or ribosome assembly, reflecting cell cycle arrest and differentiation induced by ATRA. However, a number of terms relating to neuronal development and differentiation (including “synapse organisation” and “neuron projection development”) are also present in the top 30 most significant BP terms for the TOP2B null but not the WT cells (Fig. 7 and Fig. S10), although these terms are also present for WT cells at lower significance (not shown). Two hundred and twenty-six unique genes contributed to these BP terms (i.e. neural development/function-related terms in the top 30 most statistically significant); interestingly, six of these genes that are downregulated by ATRA in BKO98 cells are significantly upregulated by ATRA in WT cells (Fig. 6C).

### Highly Expressed Genes Are More Likely to be Downregulated in TOP2B Null Cells

**Fig. 8:**
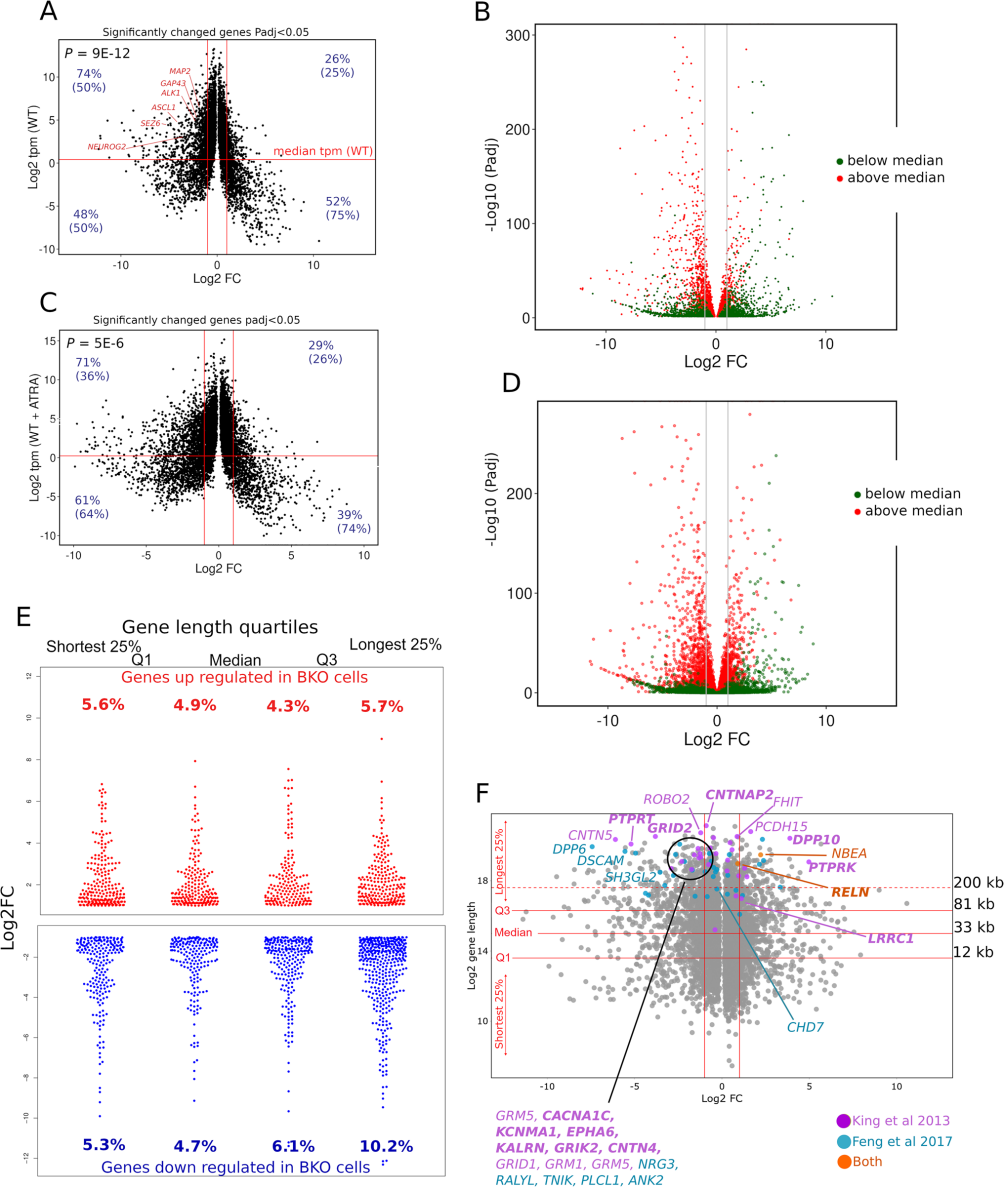
Genes downregulated in BKO98 cells are enriched for genes that are expressed at a high level in WT SH-SY5Y cells and long genes. (**A**) Volcano plot of genes differentially expressed (Padj < 0.05) between WT SH-SY5Y and BKO98 cells. Log2FC (*x* axis) is plotted against Log2 tpm (transcripts per million kilobases, *y* axis). The median tpm calculated for WT SH-SY5Y is indicated as a horizontal line. Vertical red lines indicate a Log2FC of + / − 1.0. **(B)** Volcano plot (Log10FC against -Log10 Padj) indicating genes expressed above (red) or below (green) the median tpm level in WT SH-SY5Y cells. (**C** and **D**) plots equivalent to (**A** and **B**), but data points are from ATRA-treated WT SH-SY5Y or BKO98 cells. *P* values shown for (**A**) and (**C**) are Mann–Whitney test values demonstrating significantly different Log2FC values for genes expressed at above versus below the median tmp value. For (**A**) and (**C**), figures in blue (non-bracketed) refer to the percentage of genes expressed at above or below the median tmp value that are either under or overexpressed in BKO cells. Figures in brackets are the percentages of either under or overexpressed genes that are expressed at above or below the median tmp value in wild-type cells. (**E**) Distribution and proportion of up- and downregulated protein-coding genes (Log2FC > 1.0 or <  − 1.0, Padj < 0.05) in each gene length quartile category. Numbers correspond to percentage of these genes out of all protein-coding genes expressed in the data set in each gene length category. (**F**) Plot for protein-coding genes differentially expressed between WT SH-SY5Y and BKO98 cells (Padj < 0.05) by gene length. Q1, median, and Q3 lengths are indicated by solid horizontal red lines; the dashed red line highlights a gene length of 200 kb. Genes identified by King et al. [17] as long genes affected by depletion of topoisomerase activity, and which are candidate genes for autistic spectrum disorder (ASD), are highlighted in green, and genes identified by Feng et al. [16] as downregulated in mouse CGNs upon treatment with ICRF-193 are shown in blue. Those appearing in both lists are highlighted in red. where those genes were shown to be associated with a ChIP-seq TOP2B peak [68] the names are shown in bold

We noticed that if we compared genes in our untreated SH-SY5Y RNA-seq data set according to their expression level, highly expressed genes were more likely to be downregulated in the TOP2B null cell line. Comparing genes with a relative expression level above or below the median tpm value in WT cells revealed a highly significant difference in Log2FC values between WT and BKO98 cells (Fig. 8A). Taking significantly altered genes (*P* < 0.05) with a Log2FC of > 1 or <  − 1 (i.e. in the quadrants bounded by the red lines in Fig. 8A), 74% of genes expressed above the median tpm were downregulated, and 26% were upregulated. For genes expressed below the median tpm, the proportions were 48% downregulated and 52% upregulated (Fig. 8A). The asymmetry is also visualised in the volcano plot in Fig. 8B which includes all genes, coloured according to their expression level in WT cells. This effect was also observed when comparing ATRA-treated WT cells to ATRA-treated BKO98 cells although it was less pronounced (Fig. 8C and D). Genes expressed at a relatively high level in WT cells and significantly downregulated in the TOP2B null cells include *ASCL1*, *MAP2*, *GAP43*, *ALK, SEZ6*, and *NEUROG2*.

### Long Genes Are More Likely to be Downregulated in TOP2B Null Cells

Since topoisomerases including TOP2B have previously been linked to expression of long genes in neural tissues [13, 16, 17], we examined the effect of gene length on differential gene expression between WT SH-SY5Y and BKO98 cells. When differentially expressed genes were divided by gene length quartiles, we found an overabundance of downregulated genes in the longest 25% of genes, while upregulated genes were more evenly distributed between the quartile ranges (Fig. 8E). Topoisomerases have been demonstrated to facilitate the expression of a set of autism spectrum disorder (ASD) candidate genes, many of which have very long transcripts [17]. Twenty seven of those genes are also amongst the longest 25% of genes differentially expressed between WT and TOP2B null SH-SY5Y cells (Padj < 0.05, Fig. 8F). Sixteen of these genes were significantly downregulated in BKO98 cells, 11 of those with a Log2 fold change of  > 2 (Fig. 8F). Similarly, we observed a significant overlap between genes differentially expressed in WT and TOP2B null SH-SY5Y cells and equivalent mouse genes downregulated in cultured CGN cells following treatment with the TOP2 catalytic inhibitor ICRF-193 [16] (Fig. S11). We have previously reported an overlap between genes implicated in ASD or schizophrenia and genomic localisation of TOP2B peaks derived from ChIP-seq experiments [68]. More than half of the ASD-candidate genes highlighted in Fig. 8D are associated with a TOP2B peak from this earlier study (shown in bold), including one of the longest differentially expressed transcripts, *CNTNAP2*.

## Discussion

Using differential expression analysis of RNA-seq data, we found that approximately 10% of transcripts were differentially expressed between WT and TOP2B null SH-SY5Y cells (Padj < 0.05), with a greater number of genes down- rather than upregulated (Fig. 4A, Online resource 1). Furthermore, we found an overabundance of long transcripts and more highly expressed transcripts in the set of downregulated genes (Fig. 8). The association with long genes has been reported before in mouse neuronal cells [13, 16, 17] and appears broadly consistent with the requirement for topoisomerases including TOP2B to relieve supercoiling and the resulting torsional stress that result from transcriptional elongation along the double-stranded DNA template, especially of very long genes [4, 5]. However, not all very long genes were affected in the TOP2B null cells. For example, while *CNTNAP2*, *ROBO2*, and *DPP6* were downregulated in TOP2B null cells, other long genes such as *FHIT*, *NBEA*, and *RELN* were expressed at a higher level in BKO cells (Fig. 8F). While there was a significant overlap between very long genes downregulated in our data set and Top2b-dependent genes reported in murine studies (Fig. 8F), species or cell-type differences also appear to be involved. For example, inhibition or knockdown of Top2b suppressed the expression of *Fhit*, *Nbea*, and *Reln* in murine CNS-derived cells [13, 16, 17], but as described above, *FHIT*, *NBEA*, and *RELN* were expressed at elevated levels in TOP2B null SH-SY5Y cells (Fig. 8F). Notably, *ASCL1/HASH1* and *NEUROG2* (not long genes), two genes that are key to neural specification [69], were significantly downregulated in TOP2B null cells, while *HES1*, a negative regulator of *ASCL1/HASH1* transcription, was upregulated (Fig. 4A, Table S3). Although our results cannot determine whether TOP2B has a direct effect on genes, such as *ASCL1* or *NEUROG2*, changes in the expression of these genes might be expected to have downstream effects in the expression of neural specification and maintenance of the N-type phenotype that were observed (Table S4). We also observed that genes more highly expressed in WT SH-SY5Y cells, which included *ASCL1* and *NEUROG2*, were also more likely to be downregulated in TOP2B null cells. This may be related to a requirement for topoisomerases, particularly TOP2 to relive transcription driven supercoiling that may otherwise impede transcriptional processes. Notably, studies with psoralen crosslinking to quantify supercoiling combined with topoisomerase inhibitors highlight a requirement for TOP2 specifically at highly expressed genes [5, 70, 71].

The N-type (adrenergic) and S-type (mesenchymal) neuroblastoma cells are characterised by divergent gene expression profiles that have been reported to be driven by super-enhancer associated transcription factor networks [41–43]. We noted that while the expression profile of the WT SH-SY5Y cell line places it into the adrenergic category [42], gene expression changes observed in the TOP2B null cells including upregulation of *NOTCH* family members and downregulation of *TH*, *NEFL*, and *ALK*, are consistent with a gain of mesenchymal properties (Fig. 5B) and reduction in adrenergic expression signatures*.* Adrenergic identity is associated with *PHOX2B*, *HAND2*, and *GATA3* expression; our data showed moderate downregulation of PHOX2B (Log2FC − 0.50), while *GATA3* and *HAND2 *were modestly upregulated. Notably, a number of the genes that were upregulated in the TOP2B null SH-SY5Y cells encode transcription factors associated with the MES super-enhancer network [42]; these include *NOTCH1* (Log2FC 3.1), *NOTCH2* (Log2FC 1.4), *NOTCH3* (Log2FC 1.9), *NOTCH4* (Log2FC 0.52), the NOTCH target *HES1* (Log2FC 3.5), and *YAP1* (Log2FC 3.8), as well as mesenchymal cell markers *VIM*, *SNAI2*, and *FN1* [41, 42] (Fig. 5B). Forced expression of NOTCH intracellular domains reprograms SH-SY5Y cells towards the mesenchymal phenotype [41]. Thus, upregulation of NOTCH signalling in TOP2B null cells may account for some or all of the MES phenotype-related changes observed. For example, NOTCH-mediated upregulation of the transcriptional repressor *HES1*, an established NOTCH target gene, would result in the observed downregulation of the neural specification factor *ASCL1*. This raises the question of whether *NOTCH *family members are directly regulated by TOP2B. Examination of published glioma ChIP-seq data [62] revealed TOP2B binding peaks in the regulatory regions or at internal positions of some, but not all genes discussed above. For example, TOP2B binding is present in the promoter regions of both *ASCL1* and *HES1* as well as *NOTCH1* and *NOTCH2*, and an internal TOP2B/CTCF binding peak is found in *NOTCH3* (Figs. 5E and S8). With the caveat that this TOP2B ChIP-seq data was derived from a non-neuroblastoma cell line, this is consistent with a requirement for localised TOP2B activity for correct expression of these genes. Notably, not all genes associated with the MES super-enhancer network are associated with TOP2B promoter binding in this data set, for example there was no evidence for TOP2B binding for *YAP1*.

In addition, TOP2B is a component of the TLE repressor complex that cooperates with HES1 to repress *ASCL1* in neural stem cells. The TLE1 complex is replaced by a coactivator complex in a PARP-1-dependent manner during *ASCL1* transcriptional activation [72]. This raises the possibility that TOP2B may be involved in the *ASCL1* state change from repressed to activated during neuronal cell commitment, in a way that is analogous to its role in activation in response to nuclear hormones and other stimuli [27–30, 73].

RA-induced transcriptional changes were significantly altered in TOP2B null SH-SY5Y cells extending the observation that TOP2 catalytic inhibitors ICRF-193 and merbarone interfere with the induction of retinoid-responsive genes such as *CYP26A1* and *CRABP2.* However, unlike the inhibitor experiments, in which both TOP2 isoforms are targeted, RT-PCR and RNA-seq analysis of TOP2B null cell lines directly implicate TOP2B in these expression changes. Reduced induction of genes such as *CYP26A1*, *CYP26B1, CRABP2*, and *DHRS3* that have defined retinoic acid response elements (Table S2) suggests a direct involvement of TOP2B in promoter activation of these genes, as has been reported previously for transcriptional activation by retinoic acid and other nuclear hormone ligands [29–31, 74], and for other stimulation-induced transcriptional changes [27, 28, 73]. The precise nature of the requirement for TOP2B at these promoters is unclear but may go beyond typical topoisomerase activity. As an integral part of their reaction mechanism, topoisomerases introduce transient enzyme-linked strand breaks, but the above studies have suggested that longer term DSBs possibly formed as a result of trapped TOP2-DNA covalent complexes may be required for efficient transcription state changes. Although the study reported here does not directly address this mechanistic point, the TOP2B null SH-SY5Y cell line provides a useful tool to approach this issue. Notably, not all RA-responsive genes are affected by depletion of TOP2B. *RET*, for example, is rapidly induced to an equivalent level in both WT and TOP2B null cells (Fig. 3, Table S2).

In addition, to classically RA-responsive genes such as *CYP26A1*, *CYP26B1*, *CRABP2*, and *DHRS3*, a set of genes associated with neural differentiation are induced in SH-SY5Y cells after ATRA treatment. ATRA-induced expression of some of these genes including *NTRK2*, *BCL2*, and *NTRK1* was supressed in TOP2B null cells, while other neural RA-responsive genes such as *NCAM2* are not affected (Table S3). The precise role of TOP2B in the RA-induced expression of genes such as *NTRK2* remains to be resolved, as does the mechanism behind upregulation of NOTCH family transcription factors that are associated with the observed transition towards a more mesenchymal gene expression profile. Indeed, reduced RA-stimulated neural differentiation may be a result of the weakened adrenergic transcriptional signatures in the TOP2B null cells. As supported by the glioma ChIP-seq data described above, it is likely that the presence or recruitment of TOP2B activity to the promoters of at least some of these genes is required to set their correct level of expression and response to signals, as has been observed for genes such as *Ngfr* (p75) in murine neurons [13]. In this murine study, Top2b was demonstrated to be present in the *Ngfr* promoter region in WT cells, and *Ngfr* was expressed at an elevated level in Top2b null cells. Notably, *NGFR* is also upregulated (Log2FC 1.52) in TOP2B null SH-SY5Y cells. Another example is provided by *Pax5* in the haematopoietic system where Top2b binds and is catalytically active within a broad region in the promoter and internal enhancer of the murine B cell specification factor *Pax5* and is required for correct *Pax5* expression during B cell differentiation [15]. Although the present study is limited to a single neuroblastoma cell line (SH-SY5Y), the data are consistent with and extend previous findings regarding the requirement for TOP2B for correct expression of long and of highly expressed genes, for correct expression of a subset of neural genes and for retinoid-induced gene expression. These data raise interesting mechanistic questions about TOP2B and its function in retinoid-induced transcriptional changes and for maintaining the ADRN transcriptional signature of SH-SY5Y cells. These questions could be addressed in future genome-wide analyses of TOP2B binding, cleavage, and 3D chromatin organisation in SH-SY5Y cells.

## Supplementary Information

Below is the link to the electronic supplementary material.

Supplementary file1 (XLSX 18536 KB)

Supplementary file2 (PDF 3587 KB)

## Data Availability

RNA-seq data was deposited as GSE142383 at the Gene Expression Omnibus (https://www.ncbi.nlm.nih.gov/geo/).

## References

[1] Vos SM, Tretter EM, Schmidt BH, Berger JM (2011). All tangled up: how cells direct, manage and exploit topoisomerase function. Nat Rev Mol Cell Biol.

[2] Pommier Y, Sun Y, Huang SN, Nitiss JL (2016). Roles of eukaryotic topoisomerases in transcription, replication and genomic stability. Nat Rev Mol Cell Biol.

[3] Nitiss JL (2009). DNA topoisomerase II and its growing repertoire of biological functions. Nat Rev Cancer.

[4] Liu LF, Wang JC (1987). Supercoiling of the DNA template during transcription. Proc Natl Acad Sci U S A.

[5] Ma J, Wang MD (2016). DNA supercoiling during transcription. Biophys Rev.

[6] Uuskula-Reimand L, Hou H, Samavarchi-Tehrani P, Rudan MV, Liang M, Medina-Rivera A, Mohammed H, Schmidt D, Schwalie P, Young EJ, Reimand J, Hadjur S, Gingras AC, Wilson MD (2016). Topoisomerase II beta interacts with cohesin and CTCF at topological domain borders. Genome Biol.

[7] Canela A, Maman Y, Jung S, Wong N, Callen E, Day A, Kieffer-Kwon KR, Pekowska A, Zhang H, Rao SSP, Huang SC, McKinnon PJ, Aplan PD, Pommier Y, Aiden EL, Casellas R, Nussenzweig A (2017). Genome organization drives chromosome fragility. Cell.

[8] Padget K, Pearson AD, Austin CA (2000). Quantitation of DNA topoisomerase IIalpha and beta in human leukaemia cells by immunoblotting. Leukemia.

[9] Austin CA, Lee KC, Swan RL, Khazeem MM, Manville CM, Cridland P, Treumann A, Porter A, Morris NJ, Cowell IG (2018) TOP2B: the first thirty years. Int J Mol Sci 19 (9) 10.3390/ijms19092765 10.3390/ijms19092765 PMC6163646 30223465

[10] Zandvliet DW, Hanby AM, Austin CA, Marsh KL, Clark IB, Wright NA, Poulsom R (1996). Analysis of foetal expression sites of human type II DNA topoisomerase alpha and beta mRNAs by in situ hybridisation. Biochim Biophys Acta.

[11] Zhang S, Liu X, Bawa-Khalfe T, Lu LS, Lyu YL, Liu LF, Yeh ET (2012). Identification of the molecular basis of doxorubicin-induced cardiotoxicity. Nat Med.

[12] Harkin LF, Gerrelli D, Gold Diaz DC, Santos C, Alzu'bi A, Austin CA, Clowry GJ (2016). Distinct expression patterns for type II topoisomerases IIA and IIB in the early foetal human telencephalon. J Anat.

[13] Tiwari VK, Burger L, Nikoletopoulou V, Deogracias R, Thakurela S, Wirbelauer C, Kaut J, Terranova R, Hoerner L, Mielke C, Boege F, Murr R, Peters AH, Barde YA, Schubeler D (2012). Target genes of topoisomerase IIbeta regulate neuronal survival and are defined by their chromatin state. Proc Natl Acad Sci U S A.

[14] Yang X, Li W, Prescott ED, Burden SJ, Wang JC (2000). DNA topoisomerase IIbeta and neural development. Science.

[15] Broderick L, Yost S, Li D, McGeough MD, Booshehri LM, Guaderrama M, Brydges SD, Kucharova K, Patel NC, Harr M, Hakonarson H, Zackai E, Cowell IG, Austin CA, Hugle B, Gebauer C, Zhang J, Xu X, Wang J, Croker BA, Frazer KA, Putnam CD, Hoffman HM (2019). Mutations in topoisomerase IIbeta result in a B cell immunodeficiency. Nat Commun.

[16] Feng W, Kawauchi D, Korkel-Qu H, Deng H, Serger E, Sieber L, Lieberman JA, Jimeno-Gonzalez S, Lambo S, Hanna BS, Harim Y, Jansen M, Neuerburg A, Friesen O, Zuckermann M, Rajendran V, Gronych J, Ayrault O, Korshunov A, Jones DT, Kool M, Northcott PA, Lichter P, Cortes-Ledesma F, Pfister SM, Liu HK (2017). Chd7 is indispensable for mammalian brain development through activation of a neuronal differentiation programme. Nat Commun.

[17] King IF, Yandava CN, Mabb AM, Hsiao JS, Huang HS, Pearson BL, Calabrese JM, Starmer J, Parker JS, Magnuson T, Chamberlain SJ, Philpot BD, Zylka MJ (2013). Topoisomerases facilitate transcription of long genes linked to autism. Nature.

[18] Nur-E-Kamal A, Meiners S, Ahmed I, Azarova A, Lin C-p, Lyu YL, Liu LF (2007). Role of DNA topoisomerase IIβ in neurite outgrowth. Brain Res.

[19] Heng X, Jin G, Zhang X, Yang D, Zhu M, Fu S, Li X, Le W (2012). Nurr1 regulates Top IIβ and functions in axon genesis of mesencephalic dopaminergic neurons. Mol Neurodegener.

[20] Isik S, Zaim M, Yildiz MT, Negis Y, Kunduraci T, Karakas N, Arikan G, Cetin G (2015). DNA topoisomerase IIbeta as a molecular switch in neural differentiation of mesenchymal stem cells. Ann Hematol.

[21] Zaim M, Isik S (2018). DNA topoisomerase IIbeta stimulates neurite outgrowth in neural differentiated human mesenchymal stem cells through regulation of Rho-GTPases (RhoA/Rock2 pathway) and Nurr1 expression. Stem Cell Res Ther.

[22] Li Y, Hao H, Tzatzalos E, Lin R-K, Doh S, Liu LF, Lyu YL, Cai L (2014). Topoisomerase IIbeta is required for proper retinal development and survival of postmitotic cells. Biology Open.

[23] Nevin LM, Xiao T, Staub W, Baier H (2011). Topoisomerase IIbeta is required for lamina-specific targeting of retinal ganglion cell axons and dendrites. Development.

[24] Tsutsui K, Tsutsui K, Sano K, Kikuchi A, Tokunaga A (2001). Involvement of DNA topoisomerase IIbeta in neuronal differentiation. J Biol Chem.

[25] Lyu YL, Wang JC (2003). Aberrant lamination in the cerebral cortex of mouse embryos lacking DNA topoisomerase IIbeta. Proc Natl Acad Sci U S A.

[26] Edmond M, Hanley O, Philippidou P (2017) Topoisomerase IIbeta Selectively regulates motor neuron identity and peripheral connectivity through Hox/Pbx-dependent transcriptional programs. eNeuro 4 (6) 10.1523/ENEURO.0404-17.2017 10.1523/ENEURO.0404-17.2017 PMC5779120 29379870

[27] Bunch H, Lawney BP, Lin Y-F, Asaithamby A, Murshid A, Wang YE, Chen BPC, Calderwood SK (2015) Transcriptional elongation requires DNA break-induced signalling. Nat Commun 6 10.1038/ncomms10191 10.1038/ncomms10191 PMC4703865 26671524

[28] Madabhushi R, Gao F, Pfenning Andreas R, Pan L, Yamakawa S, Seo J, Rueda R, Phan TX, Yamakawa H, Pao P-C, Stott Ryan T, Gjoneska E, Nott A, Cho S, Kellis M, Tsai L-H (2015). Activity-Induced DNA breaks govern the expression of neuronal early-response genes. Cell.

[29] Ju BG, Lunyak VV, Perissi V, Garcia-Bassets I, Rose DW, Glass CK, Rosenfeld MG (2006). A topoisomerase IIβ-mediated dsDNA break required for regulated transcription. Science.

[30] Haffner MC, Aryee MJ, Toubaji A, Esopi DM, Albadine R, Gurel B, Isaacs WB, Bova GS, Liu W, Xu J, Meeker AK, Netto G, De Marzo AM, Nelson WG, Yegnasubramanian S (2010). Androgen-induced TOP2B-mediated double-strand breaks and prostate cancer gene rearrangements. Nat Genet.

[31] McNamara S, Wang H, Hanna N, Miller WH (2008). Topoisomerase IIβ negatively modulates retinoic acid receptor α function: a novel mechanism of retinoic acid resistance. Mol Cell Biol.

[32] Brown AM, Riddoch FC, Robson A, Redfern CP, Cheek TR (2005). Mechanistic and functional changes in Ca2+ entry after retinoic acid-induced differentiation of neuroblastoma cells. Biochem J.

[33] Riddoch FC, Rowbotham SE, Brown AM, Redfern CP, Cheek TR (2005). Release and sequestration of Ca2+ by a caffeine- and ryanodine-sensitive store in a sub-population of human SH-SY5Y neuroblastoma cells. Cell Calcium.

[34] Bell N, Hann V, Redfern CPF, Cheek TR (2013). Store-operated Ca2 + entry in proliferating and retinoic acid-differentiated N- and S- type neuroblastoma cells. Biochimica et Biophysica Acta (BBA) Molecular Cell Research.

[35] Kovalevich J, Langford D (2013). Considerations for the use of SH-SY5Y neuroblastoma cells in neurobiology. Methods Mol Biol.

[36] Ciccarone V, Spengler BA, Meyers MB, Biedler JL, Ross RA (1989). Phenotypic diversification in human neuroblastoma cells: expression of distinct neural crest lineages. Cancer Res.

[37] Ross RA, Spengler BA (2007). Human neuroblastoma stem cells. Semin Cancer Biol.

[38] Biedler JL, Roffler-Tarlov S, Schachner M, Freedman LS (1978). Multiple neurotransmitter synthesis by human neuroblastoma cell lines and clones. Cancer Res.

[39] Walton JD, Kattan DR, Thomas SK, Spengler BA, Guo HF, Biedler JL, Cheung NK, Ross RA (2004). Characteristics of stem cells from human neuroblastoma cell lines and in tumors. Neoplasia.

[40] Ross RA, Spengler BA, Biedler JL (1983). Coordinate morphological and biochemical interconversion of human neuroblastoma cells. J Natl Cancer Inst.

[41] van Groningen T, Akogul N, Westerhout EM, Chan A, Hasselt NE, Zwijnenburg DA, Broekmans M, Stroeken P, Haneveld F, Hooijer GKJ, Savci-Heijink CD, Lakeman A, Volckmann R, van Sluis P, Valentijn LJ, Koster J, Versteeg R, van Nes J (2019). A NOTCH feed-forward loop drives reprogramming from adrenergic to mesenchymal state in neuroblastoma. Nat Commun.

[42] van Groningen T, Koster J, Valentijn LJ, Zwijnenburg DA, Akogul N, Hasselt NE, Broekmans M, Haneveld F, Nowakowska NE, Bras J, van Noesel CJM, Jongejan A, van Kampen AH, Koster L, Baas F, van Dijk-Kerkhoven L, Huizer-Smit M, Lecca MC, Chan A, Lakeman A, Molenaar P, Volckmann R, Westerhout EM, Hamdi M, van Sluis PG, Ebus ME, Molenaar JJ, Tytgat GA, Westerman BA, van Nes J, Versteeg R (2017). Neuroblastoma is composed of two super-enhancer-associated differentiation states. Nat Genet.

[43] Boeva V, Louis-Brennetot C, Peltier A, Durand S, Pierre-Eugene C, Raynal V, Etchevers HC, Thomas S, Lermine A, Daudigeos-Dubus E, Geoerger B, Orth MF, Grunewald TGP, Diaz E, Ducos B, Surdez D, Carcaboso AM, Medvedeva I, Deller T, Combaret V, Lapouble E, Pierron G, Grossetete-Lalami S, Baulande S, Schleiermacher G, Barillot E, Rohrer H, Delattre O, Janoueix-Lerosey I (2017). Heterogeneity of neuroblastoma cell identity defined by transcriptional circuitries. Nat Genet.

[44] Gautier M, Thirant C, Delattre O, Janoueix-Lerosey I (2021) Plasticity in neuroblastoma cell identity defines a noradrenergic-to-mesenchymal transition (NMT). Cancers (Basel) 13(12) 10.3390/cancers13122904 10.3390/cancers13122904 PMC8230375 34200747

[45] Teppola H, Sarkanen JR, Jalonen TO, Linne ML (2016). Morphological differentiation towards neuronal phenotype of SH-SY5Y neuroblastoma cells by estradiol, retinoic acid and cholesterol. Neurochem Res.

[46] Kaplan DR, Matsumoto K, Lucarelli E, Thiele CJ (1993). Induction of TrkB by retinoic acid mediates biologic responsiveness to BDNF and differentiation of human neuroblastoma cells. Eukaryotic Signal Transduction Group Neuron.

[47] Pezzini F, Bettinetti L, Leva FD, Bianchi M, Zoratti E, Carrozzo R, Santorelli FM, Delledonne M, Lalowski M, Simonati A (2017). Transcriptomic profiling discloses molecular and cellular events related to neuronal differentiation in SH-SY5Y neuroblastoma cells. Cell Mol Neurobiol.

[48] Oppenheimer O, Cheung NK, Gerald WL (2007). The RET oncogene is a critical component of transcriptional programs associated with retinoic acid-induced differentiation in neuroblastoma. Mol Cancer Ther.

[49] Khazeem MM, Cowell IG, Harkin LF, Casement JW, Austin CA (2020) Transcription of carbonyl reductase 1 is regulated by DNA topoisomerase II beta. FEBS Lett 10.1002/1873-3468.13904 10.1002/1873-3468.13904 32767399

[50] Patro R, Duggal G, Love MI, Irizarry RA, Kingsford C (2017). Salmon provides fast and bias-aware quantification of transcript expression. Nat Methods.

[51] Love MI, Huber W, Anders S (2014). Moderated estimation of fold change and dispersion for RNA-seq data with DESeq2. Genome Biol.

[52] Suarez-Arnedo A, Torres Figueroa F, Clavijo C, Arbelaez P, Cruz JC, Munoz-Camargo C (2020). An image J plugin for the high throughput image analysis of in vitro scratch wound healing assays. PLoS ONE.

[53] Merrill RA, Ahrens JM, Kaiser ME, Federhart KS, Poon VY, Clagett-Dame M (2004) All-trans retinoic acid-responsive genes identified in the human SH-SY5Y neuroblastoma cell line and their regulated expression in the nervous system of early embryos. Biol Chem 385 (7)10.1515/BC.2004.075 10.1515/BC.2004.075 15318809

[54] Fortune JM, Osheroff N (1998). Merbarone Inhibits the Catalytic Activity of Human Topoisomerase IIα by Blocking DNA Cleavage. J Biol Chem.

[55] Roca J, Ishida R, Berger JM, Andoh T, Wang JC (1994). Antitumor bisdioxopiperazines inhibit yeast DNA topoisomerase II by trapping the enzyme in the form of a closed protein clamp. Proc Natl Acad Sci U S A.

[56] Pahlman S, Ruusala AI, Abrahamsson L, Mattsson ME, Esscher T (1984). Retinoic acid-induced differentiation of cultured human neuroblastoma cells: a comparison with phorbolester-induced differentiation. Cell Differ.

[57] Hanada M, Krajewski S, Tanaka S, Cazals-Hatem D, Spengler BA, Ross RA, Biedler JL, Reed JC (1993). Regulation of Bcl-2 oncoprotein levels with differentiation of human neuroblastoma cells. Cancer Res.

[58] Itano Y, Ito A, Uehara T, Nomura Y (1996). Regulation of Bcl-2 protein expression in human neuroblastoma SH-SY5Y cells: positive and negative effects of protein kinases C and A, respectively. J Neurochem.

[59] Merry DE, Veis DJ, Hickey WF, Korsmeyer SJ (1994). bcl-2 protein expression is widespread in the developing nervous system and retained in the adult PNS. Development.

[60] Garcia I, Martinou I, Tsujimoto Y, Martinou JC (1992). Prevention of programmed cell death of sympathetic neurons by the bcl-2 proto-oncogene. Science.

[61] van Nes J, Chan A, van Groningen T, van Sluis P, Koster J, Versteeg R (2013). A NOTCH3 transcriptional module induces cell motility in neuroblastoma. Clin Cancer Res.

[62] Gonzalez-Buendia E, Zhao J, Wang L, Mukherjee S, Zhang D, Arrieta VA, Feldstein E, Kane JR, Kang SJ, Lee-Chang C, Mahajan A, Chen L, Realubit R, Karan C, Magnuson L, Horbinski C, Marshall SA, Sarkaria JN, Mohyeldin A, Nakano I, Bansal M, James CD, Brat DJ, Ahmed A, Canoll P, Rabadan R, Shilatifard A, Sonabend AM (2021). TOP2B enzymatic activity on promoters and introns modulates multiple oncogenes in human gliomas. Clin Cancer Res.

[63] Cheung Y-T, Lau WK-W, Yu M-S, Lai CS-W, Yeung S-C, So K-F, Chang RC-C (2009). Effects of all-trans-retinoic acid on human SH-SY5Y neuroblastoma as in vitro model in neurotoxicity research. Neurotoxicology.

[64] Yan Y-x, Zhao J-x, Han S, Zhou N-j, Jia Z-q, Yao S-j, Cao C-l, Wang Y-l, Xu Y-n, Zhao J, Yan Y-l, Cui H-x (2015). Tetramethylpyrazine induces SH-SY5Y cell differentiation toward the neuronal phenotype through activation of the PI3K/Akt/Sp1/TopoIIβ pathway. Eur J Cell Biol.

[65] Zha Y, Ding E, Yang L, Mao L, Wang X, McCarthy BA, Huang S, Ding HF (2012). Functional dissection of HOXD cluster genes in regulation of neuroblastoma cell proliferation and differentiation. PLoS ONE.

[66] Garcia-Gutierrez L, Delgado MD, Leon J (2019) MYC Oncogene contributions to release of cell cycle brakes. Genes (Basel) 10 (3) 10.3390/genes10030244 10.3390/genes10030244 PMC6470592 30909496

[67] Raudvere U, Kolberg L, Kuzmin I, Arak T, Adler P, Peterson H, Vilo J (2019). g:Profiler: a web server for functional enrichment analysis and conversions of gene lists (2019 update). Nucleic Acids Res.

[68] Manville CM, Smith K, Sondka Z, Rance H, Cockell S, Cowell IG, Lee KC, Morris NJ, Padget K, Jackson GH, Austin CA (2015). Genome-wide ChIP-seq analysis of human TOP2B occupancy in MCF7 breast cancer epithelial cells. Biol Open.

[69] Bertrand N, Castro DS, Guillemot F (2002). Proneural genes and the specification of neural cell types. Nat Rev Neurosci.

[70] Teves SS, Henikoff S (2014). Transcription-generated torsional stress destabilizes nucleosomes. Nat Struct Mol Biol.

[71] Kouzine F, Gupta A, Baranello L, Wojtowicz D, Ben-Aissa K, Liu J, Przytycka TM, Levens D (2013). Transcription-dependent dynamic supercoiling is a short-range genomic force. Nat Struct Mol Biol.

[72] Ju BG, Solum D, Song EJ, Lee KJ, Rose DW, Glass CK, Rosenfeld MG (2004). Activating the PARP-1 sensor component of the groucho/ TLE1 corepressor complex mediates a CaMKinase IIdelta-dependent neurogenic gene activation pathway. Cell.

[73] Wong RHF, Chang I, Hudak CSS, Hyun S, Kwan H-Y, Sul HS (2009). A role of DNA-PK for the metabolic gene regulation in response to insulin. Cell.

[74] Trotter KW, King HA, Archer TK (2015). Glucocorticoid receptor transcriptional activation via the BRG1-dependent recruitment of TOP2β and Ku70/86. Mol Cell Biol.

[75] Thorvaldsdottir H, Robinson JT, Mesirov JP (2013). Integrative genomics viewer (IGV): high-performance genomics data visualization and exploration. Brief Bioinform.

[76] Zimmerman MW, Liu Y, He S, Durbin AD, Abraham BJ, Easton J, Shao Y, Xu B, Zhu S, Zhang X, Li Z, Weichert-Leahey N, Young RA, Zhang J, Look AT (2018). MYC drives a subset of high-risk pediatric neuroblastomas and is activated through mechanisms including enhancer hijacking and focal enhancer amplification. Cancer Discov.

[77] Gartlgruber M, Sharma AK, Quintero A, Dreidax D, Jansky S, Park Y-G, Kreth S, Meder J, Doncevic D, Saary P, Toprak UH, Ishaque N, Afanasyeva E, Wecht E, Koster J, Versteeg R, Grünewald TGP, Jones DTW, Pfister SM, Henrich K-O, van Nes J, Herrmann C, Westermann F (2021). Super enhancers define regulatory subtypes and cell identity in neuroblastoma. Nat Cancer.

