# Supplementary material for: TOP2B Is Required to Maintain the Adrenergic Neural Phenotype and for ATRA-Induced Differentiation of SH-SY5Y Neuroblastoma Cells
Source: Mol Neurobiol. 2022 Jul 13;59(10):5987–6008. doi: 10.1007/s12035-022-02949-6 (PMC9463316; doi:10.1007/s12035-022-02949-6)
Supplement: Supplementary file 2 — Supplementary file2 (PDF 3587 KB) [file 12035_2022_2949_MOESM2_ESM.pdf]

Supplementary material

Article title: TOP2B is required to maintain the adrenergic neural phenotype and for ATRA-induced differentiation of SH-SY5Y neuroblastoma cells.

Journal name: Molecular Neurobiology

Authors name and affiliations:

Mushtaq M. Khazeem[1], John W. Casement[2], George Schlossmacher[1], Niall S. Kenneth[1,3], Nielda K. Sumbung[1], Janice Yuen Tung Chan[1], Jade F. McGow[1], Ian G. Cowell[1]*, and Caroline A. Austin[1]*

***Corresponding authors**

Prof. Caroline A. Austin
caroline.austin@ncl.ac.uk. ORCID https://orcid.org/0000-0002-1921-5947

Dr Ian G. Cowell
ian.cowell@ncl.ac.uk. ORCID https://orcid.org/0000-0002-8606-0447

Other authors
MMK: https://orcid.org/0000-0003-2948-8225
NSK: https://orcid.org/0000-0001-8528-1021
GS: https://orcid.org/0000-0001-7713-5729
NZS
JYTC: https://orcid.org/0000-0001-9203-2699
JM

Addresses:

1. Biosciences Institute, The Faculty of Medical Sciences, Newcastle University, Newcastle upon Tyne NE2 4HH, UK
2. Bioinformatics Support Unit, The Faculty of Medical Sciences, Newcastle University, Newcastle upon Tyne NE2 4HH, UK
3. Current address: National Center of Hematology, Mustansiriyah University, Baghdad, Iraq.
4. Current address: Institute of Systems, Molecular and Integrative Biology, University of Liverpool, Liverpool L69 7ZB, UK.

Supplemental Figures, Tables and Legends

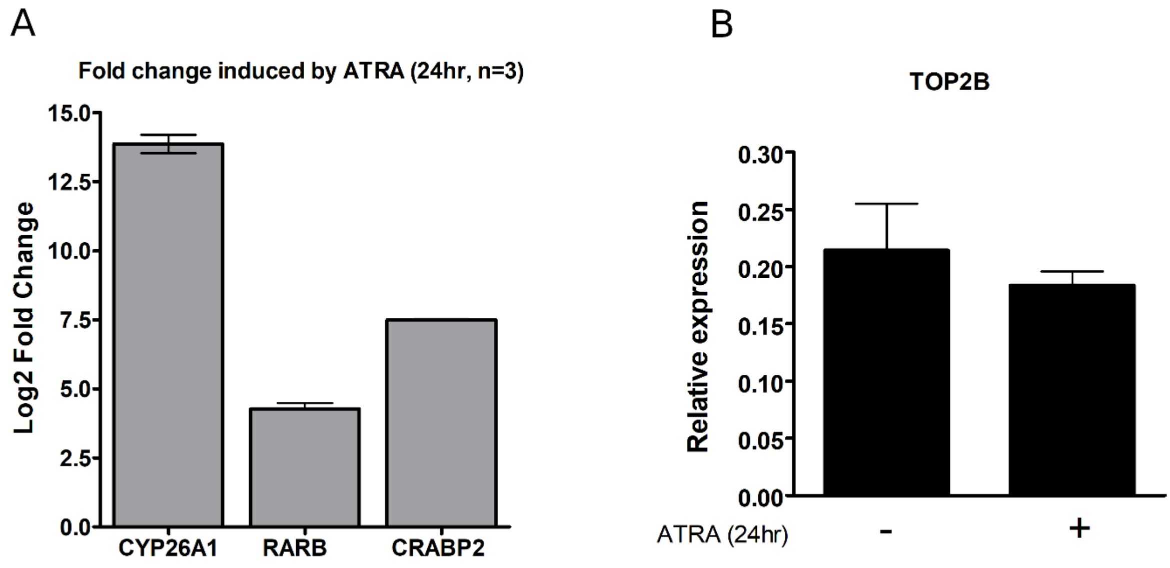

Figure S1. SH-SY5Y respond to ATRA. Cells were treated with 1μM ATRA or solvent control for 24 hours before collecting cells for RT-PCR analysis. **(A)** Induction of *CYP26A1*, *RARB* and *CRABP2* expression. **(B)** TOP2B expression is not affected by ATRA treatment. Data are expressed as expression relative to that of PP1A, were obtained from three biological replicates and are shown as mean values ± SEM.

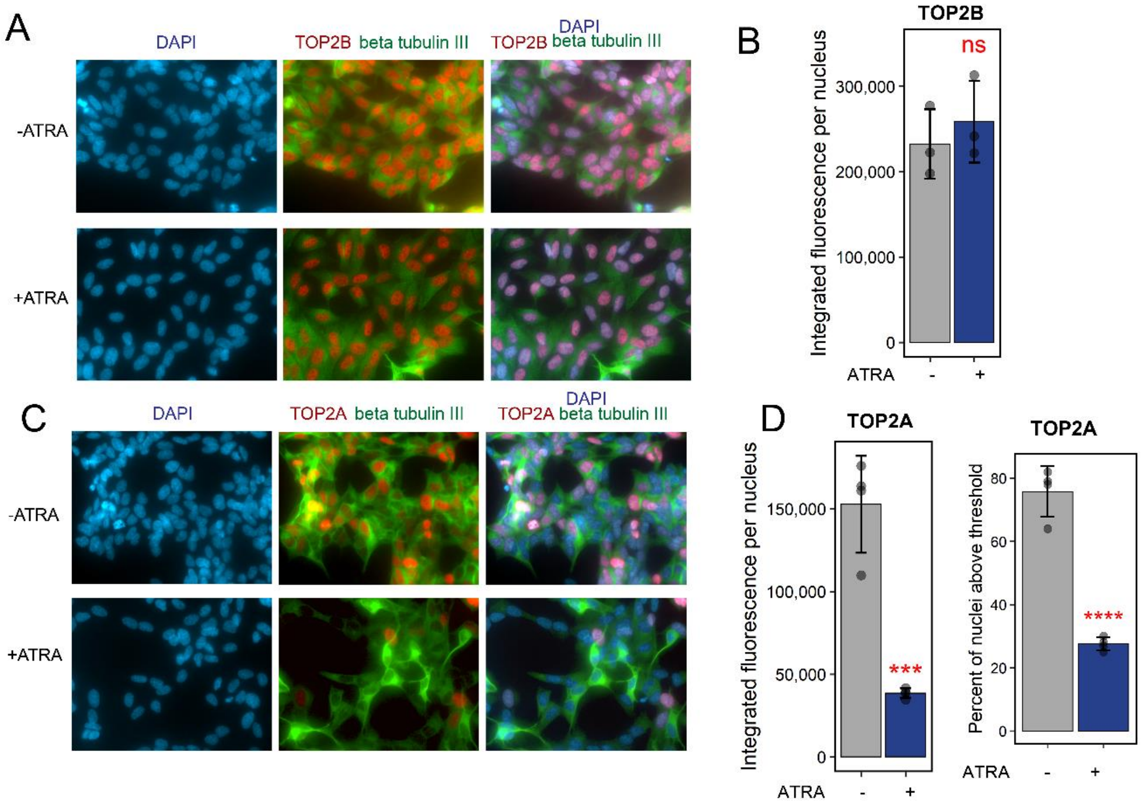

Figure S2. Expression of TOP2B and TOP2A in SH-SY5Y cells before and after ATRA-induced differentiation. Cells were cultured for 5 days in the presence of 1µM ATRA or solvent control. **(A)** Immunofluorescence analysis (40X objective) was carried out of using anti-beta-tubulin III (cytoplasmic neuronal marker, green, 2G10) and anti-TOP2B (red, 4555, top). **(B)** Relative TOP2B expression using quantitative immunofluorescence with anti-TOP2B-MAB6346. Data are expressed as the mean of the median fluorescence per nucleus values obtained for three replicates. **(C)** TOP2A immunofluorescence images as for (A) but employing anti-TOP2A 4566. **(D)** Quantitation of TOP2A expression. Left relative expression per cell (as in (B)); right, percentage of cells that strongly expressed TOP2A before and after ATRA treatment. The threshold for “strong expression” was taken as the mean of the lower quartile value for control (no ATRA) cell replicas. Statistical analysis was performed by t-test (*** = *P*<0.001, **** = *P*<0.0001).

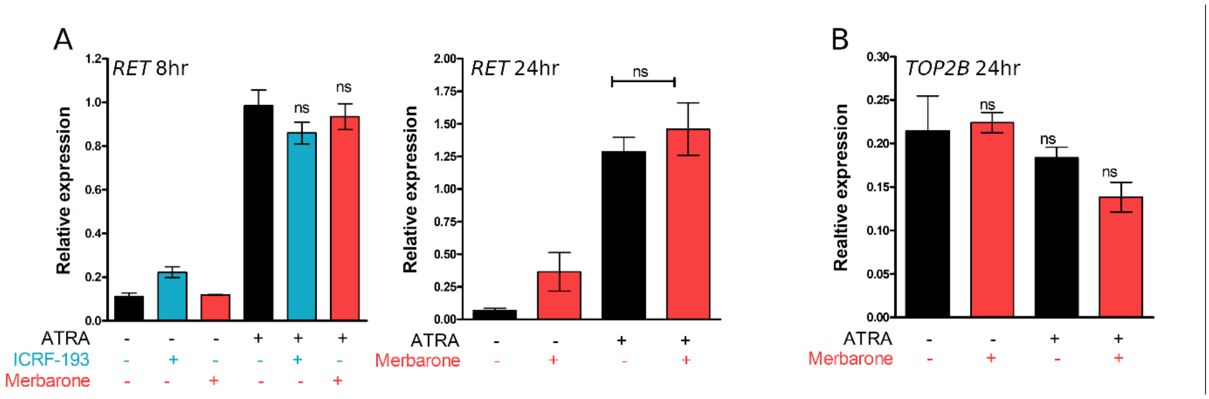

Figure S3. TOP2 catalytic inhibitors do not significantly affect ATRA-induced expression of *RET*, nor the expression of TOP2B. Relative expression for **(A)** *RET* and **(B)** *TOP2B.* See legend to Fig. 1 for details

A

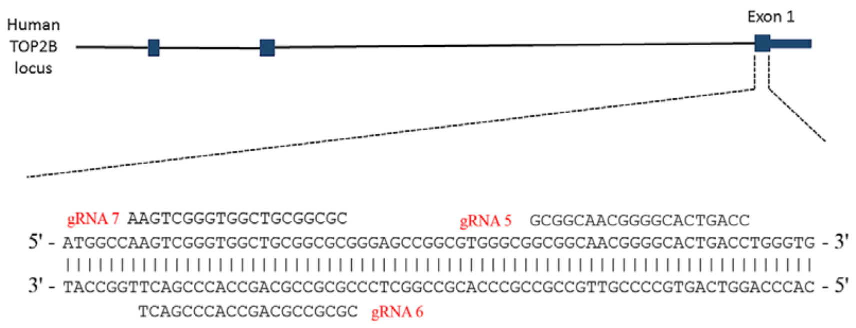

B

Clone	gRNA	Mutation
70	5	Wild: GCGGCGGCAACGGGGCACTGACCTGGGTGGTAAGTGGCTGG<br>Clone: GCGGCGGCAAC-----------CTGGGTGGTAAGTGGCTGG
98	6	Wild: AGGCACTCGCCATGGCCAAGTCGGGTGGCTGCGGCGCGGGAG<br>Clone: AGGCACTCGCCATGGCCAA-----GTGGCTGCGGCGCGGGAG
129	7	Wild: TCGCCATGGCCAAGTCGGGTGGCTGCGGCGCGGGAGCCG<br>Clone: TCGCCATGGCCAAGTCGGGTGGCTGC-----GGGAGCCG
CRISPR-*Cas9* introduced TOP2B silencing mutations		

C

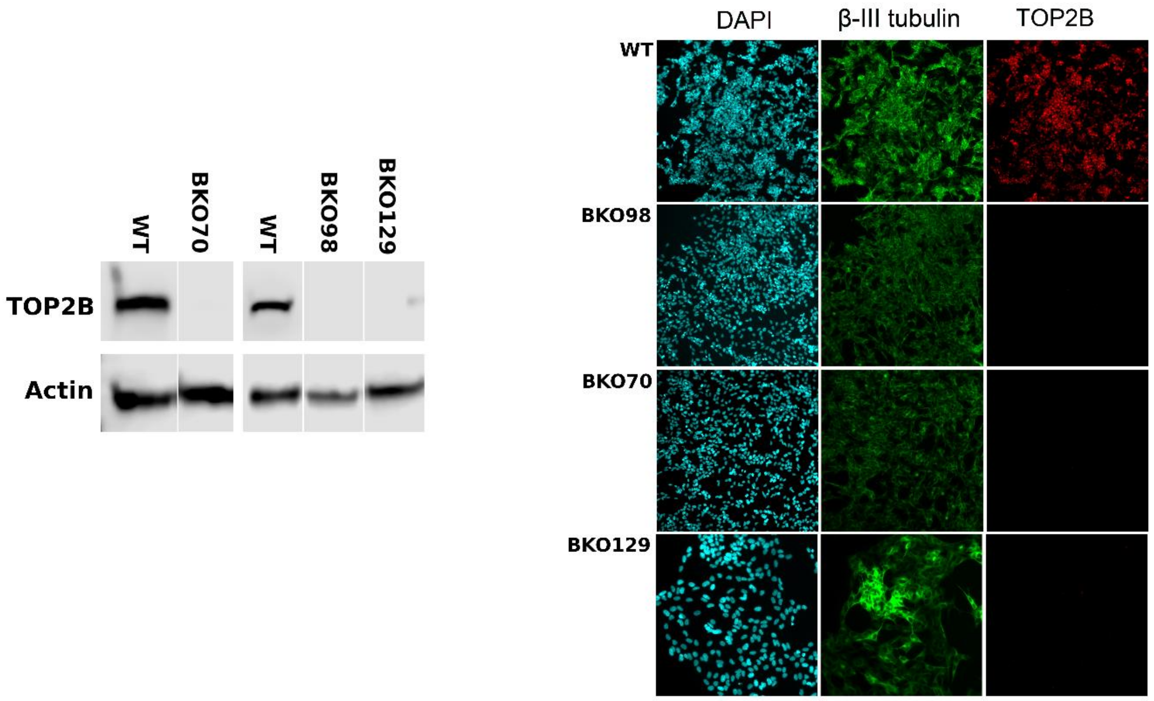

Figure S4. TOP2B CRISPR-*Cas9* guide RNAs and verification of TOP2B null SH-SY5Y clones. (A) position of guide RNAs within TOP2B exon 1. **(B)** DNA sequencing results verifying homozygous frame shift mutations in TOP2B$^{-/-}$ clones BKO70, BKO98 and BKO129. **(C)** Western blot and immunofluorescence verification of TOP2B null phenotype.

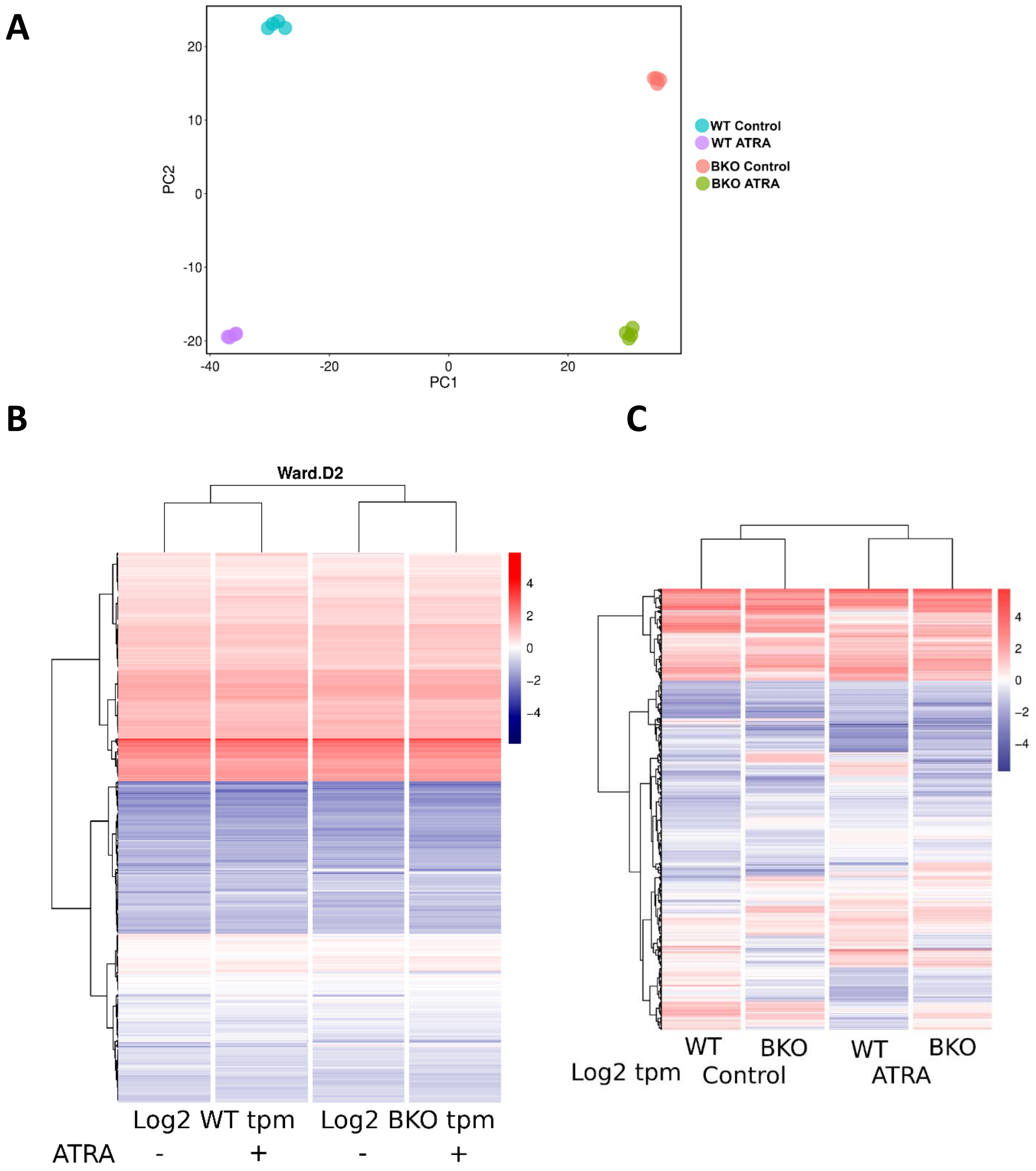

Figure S5. RNA-seq data characteristics. (**A**) PCA plot from DESeq2 illustrating tight clustering of the four RNA biological replica samples. ATRA treatment was for 10µM ATRA in ethanol for 24hr, control cells were treated with an equivalent volume of ethanol. **(B)** Heatmap comparing relative expression levels (tpm values from four replicates) from each of the four conditions for genes. **(C)** Heatmap as for (B) but including only genes whose expression changes in WT cells upon ATRA treatment with a Log2FC > 2 or <-2.

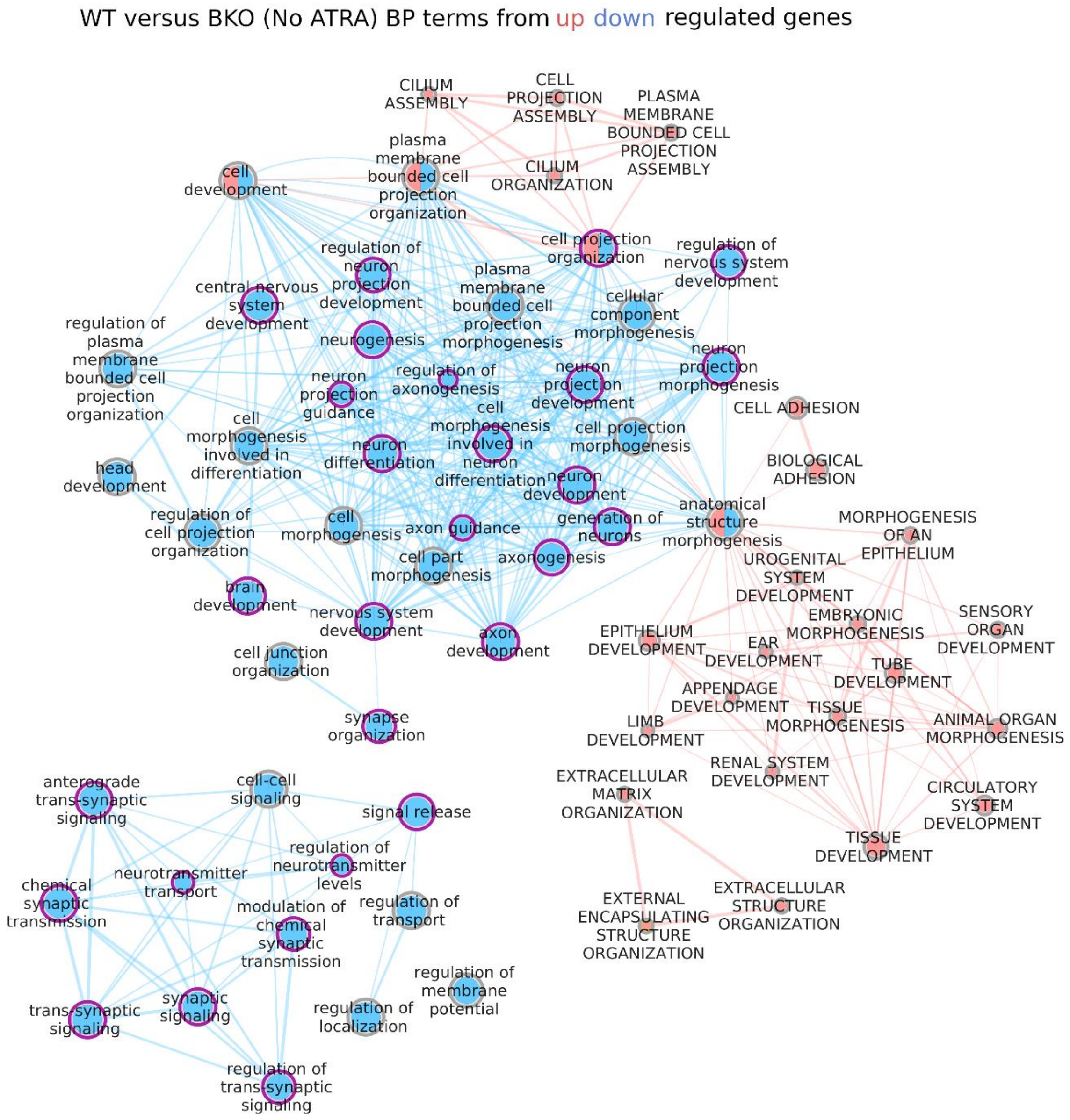

Figure S6. Functional interaction network of GO:Biological Process terms associated with genes up- (red) or down- (blue) regulated >1.5 X in TOP2B null (BKO98) versus WT SH-SY5Y cells. Terms related primarily with neuronal development or function are circled in purple.

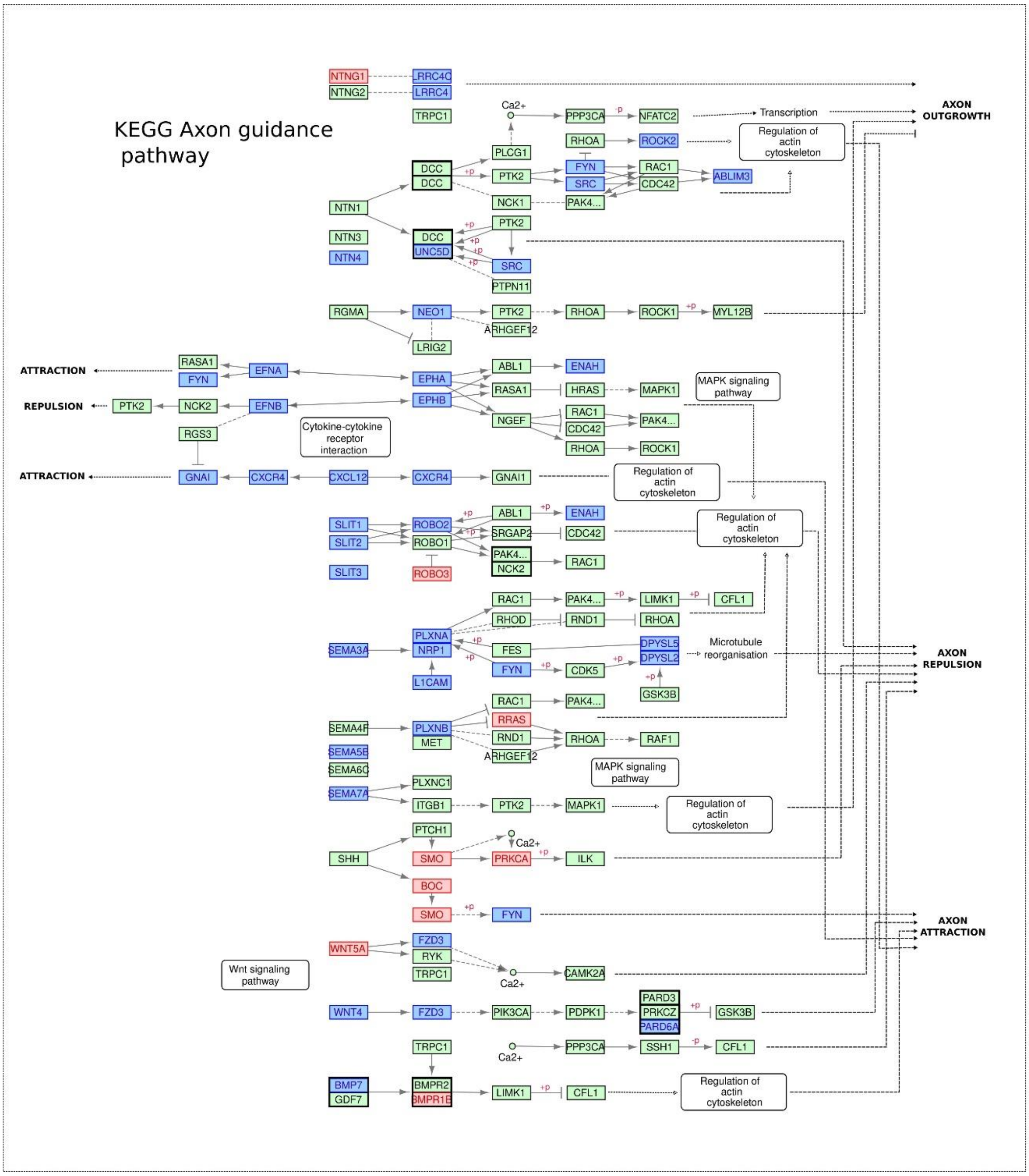

Figure S7. KEGG axon guidance pathway. Genes down regulated in TOP2B null SH-SY5Y cells are highlighted in blue, and those upregulated are highlighted in red.

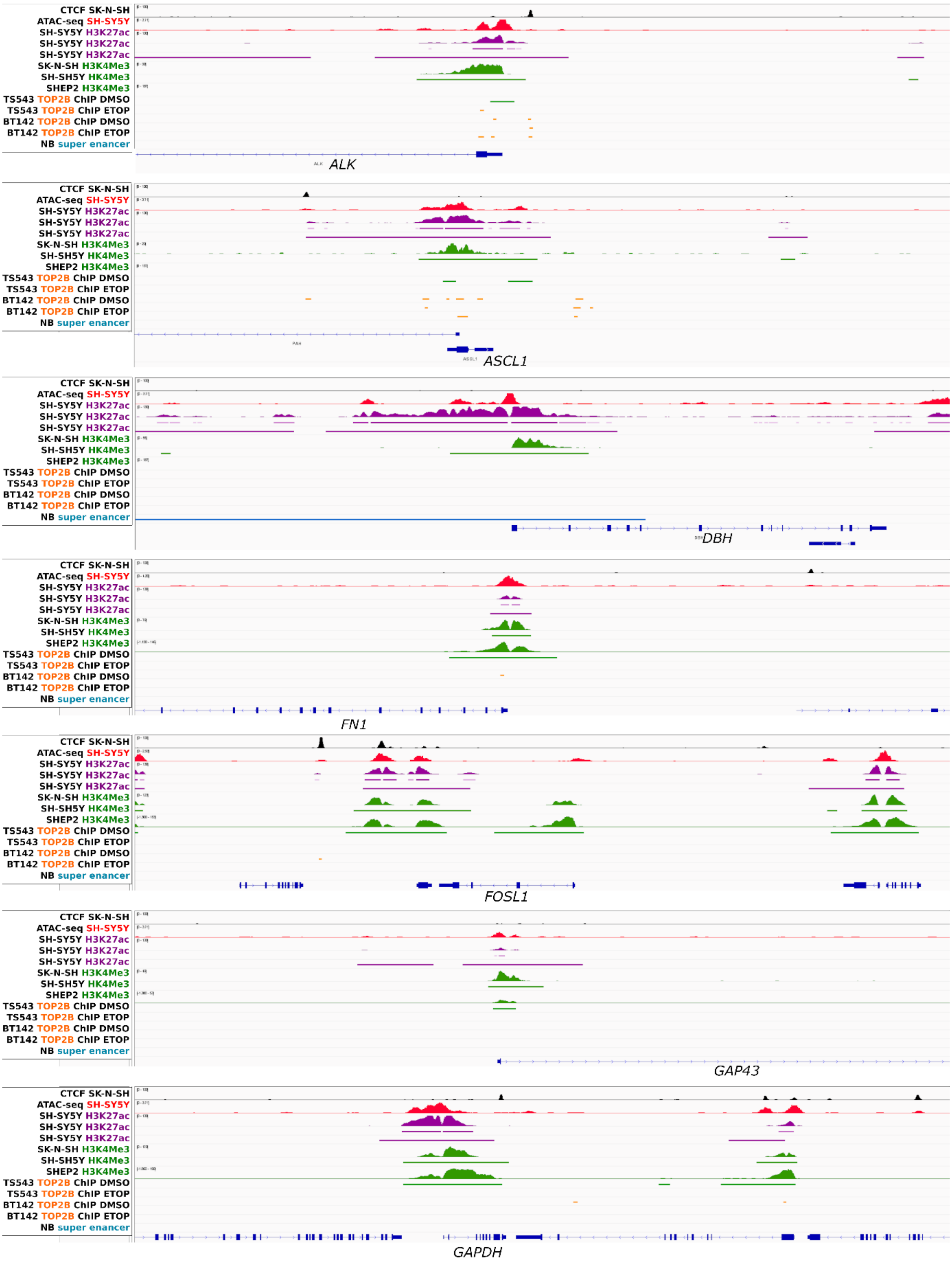

Fig. S8 continued on next page

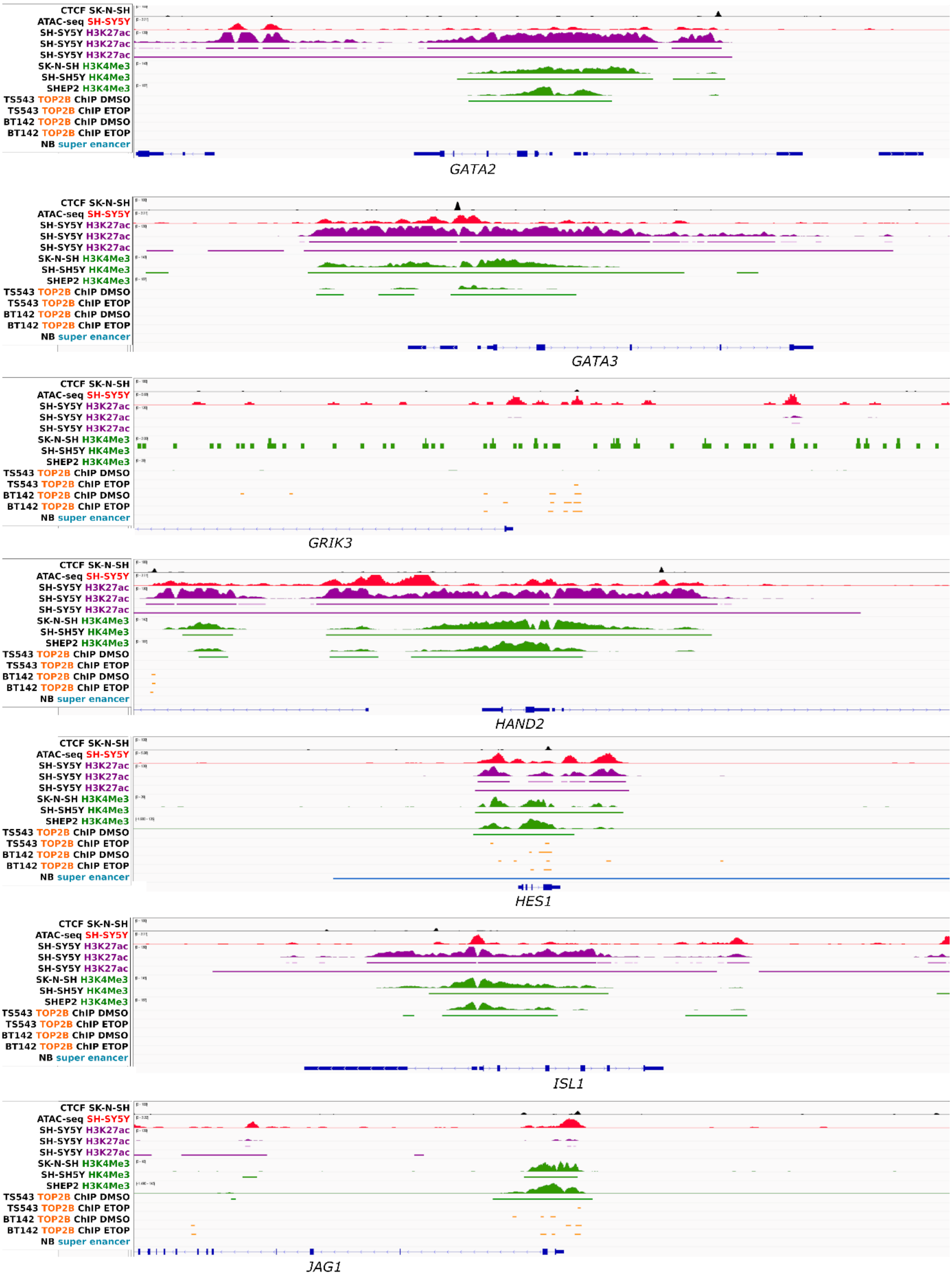

Fig. S8 continued on next page

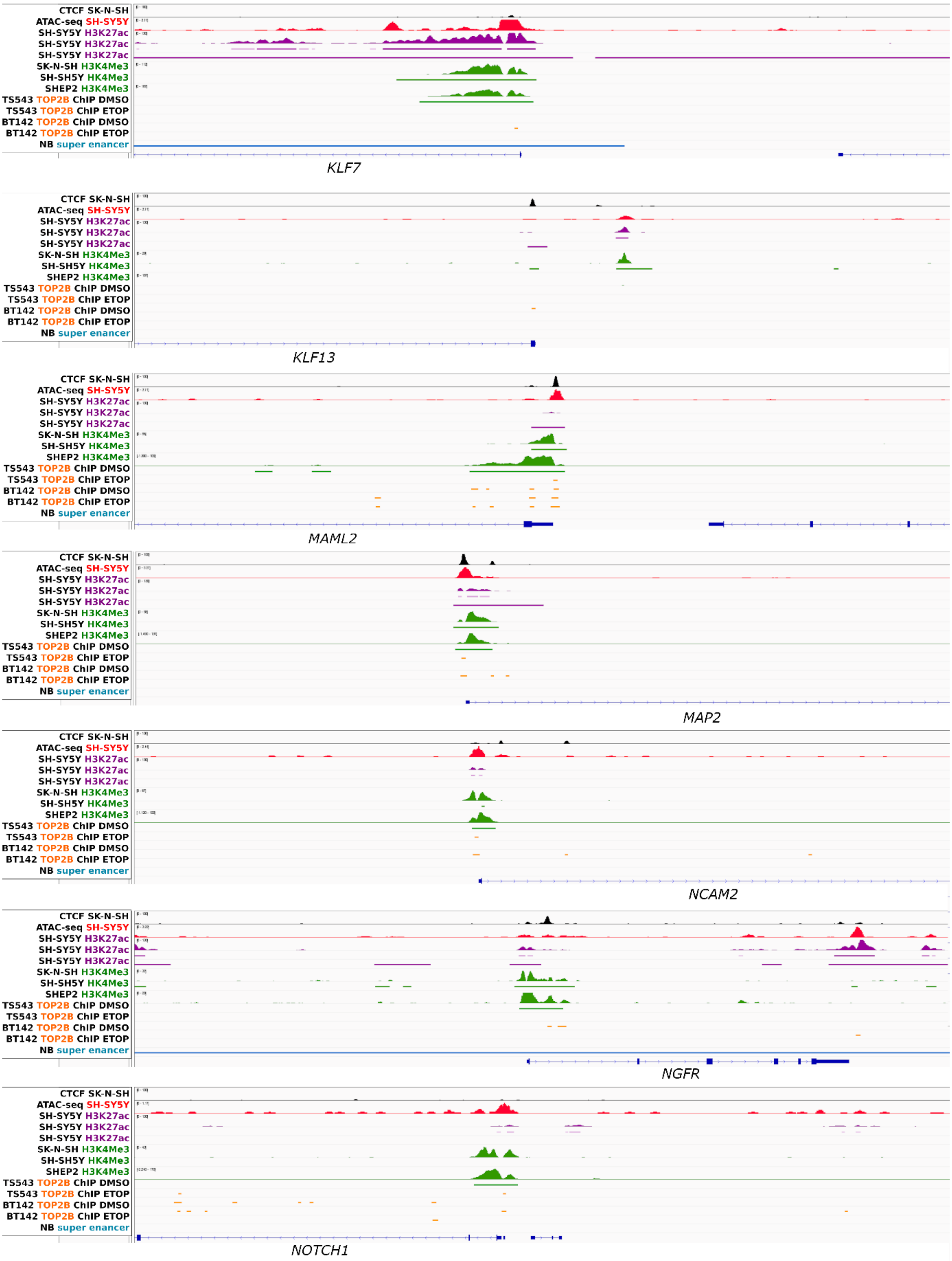

Fig. S8 continued on next page

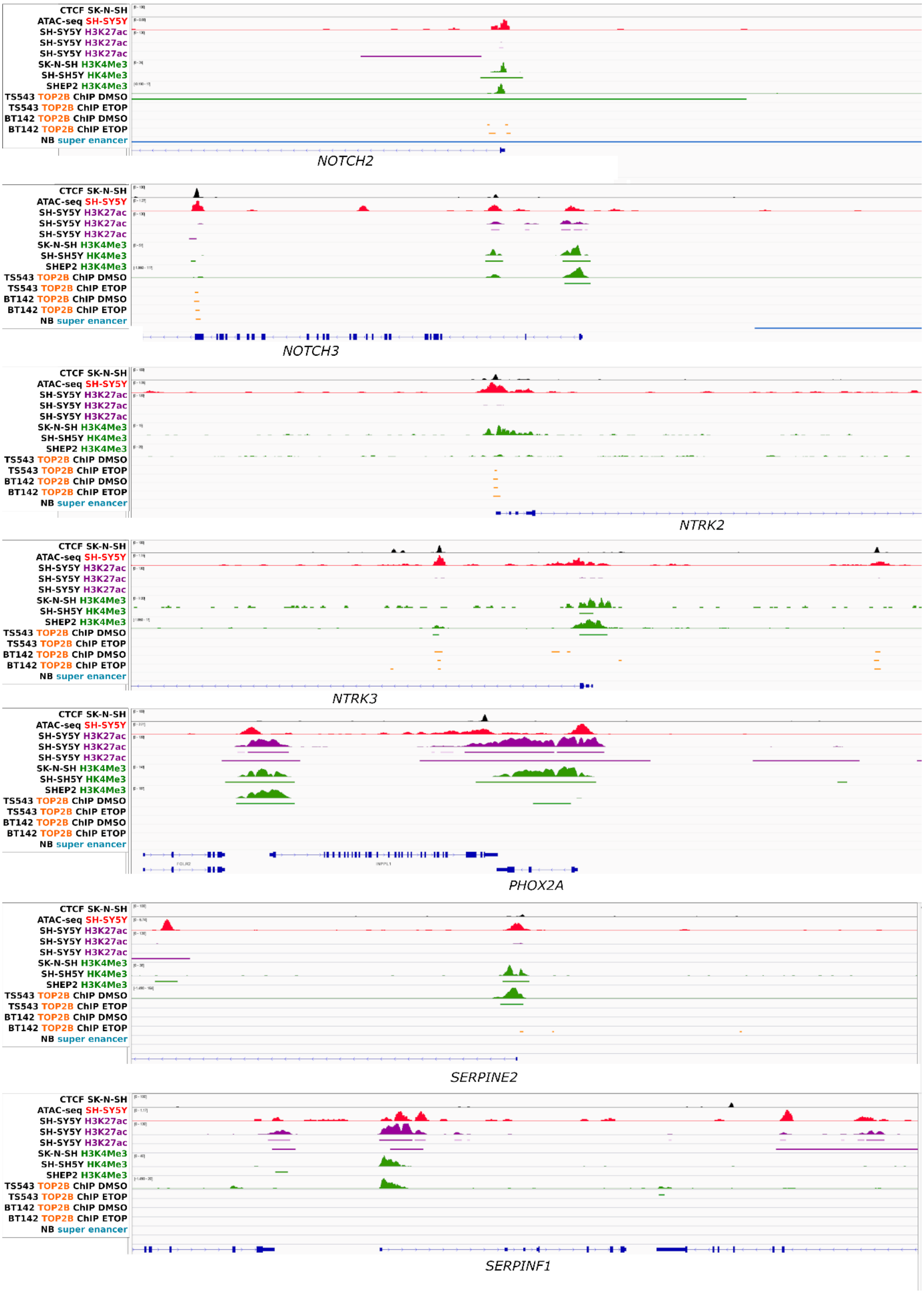

Fig. S8 continued on next page

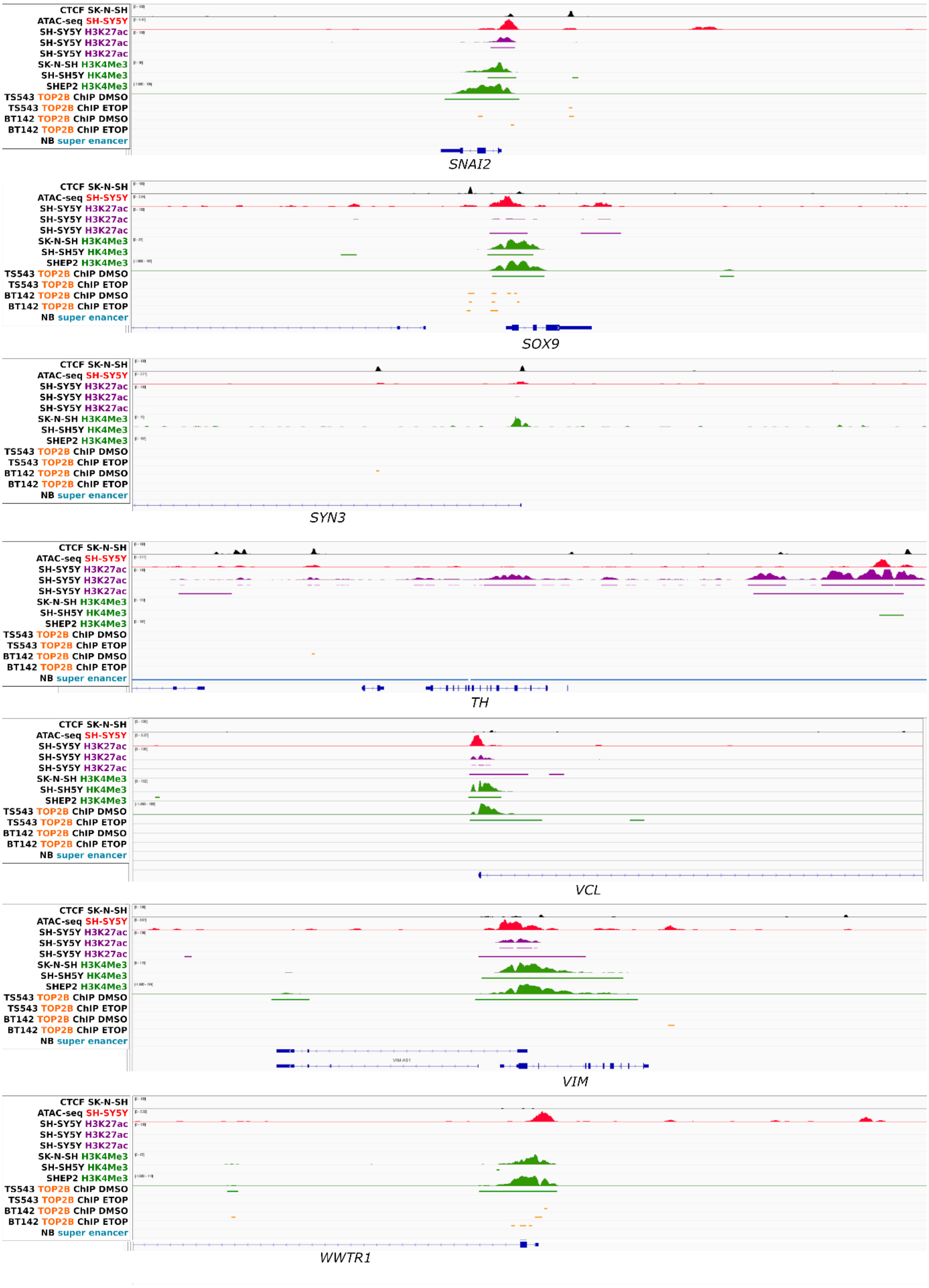

Fig. S8 legend on next page

Figure S8 IGV plots of TOP2B peaks. Epigenetic profiles for a number of genes of interest, spanning a 50 kb window centred on the predominant transcription initiation site of each gene. Data was plotted using the Integrated Genome Viewer (IGV, Broad Institute)[1] using the following data sources (GEO accession and citation) : SK-N-SH CTCF ChIP-seq, GSM2038347; SH-SY5Y ATAC-seq (open chromatin), GSM2700775 [2]; SH-SY5Y H3K27ac (active enhancers and promoters), GSM3676080 [3]; SK-N-SH ,SH-SY5Y and SH-EP H3K4Me3 (active promoter), GSM2038349, GSM2419933, GSM2419932 [4,5]; TOP2B ChIP-seq data from TS543 and BT142 glioma cell lines in the absence (DMSO) or presence of etoposide (ETO), GSM3904399, GSM3904400, GSM3904401, GSM3904402 [6], Neuroblastoma super-enhancers [5] .

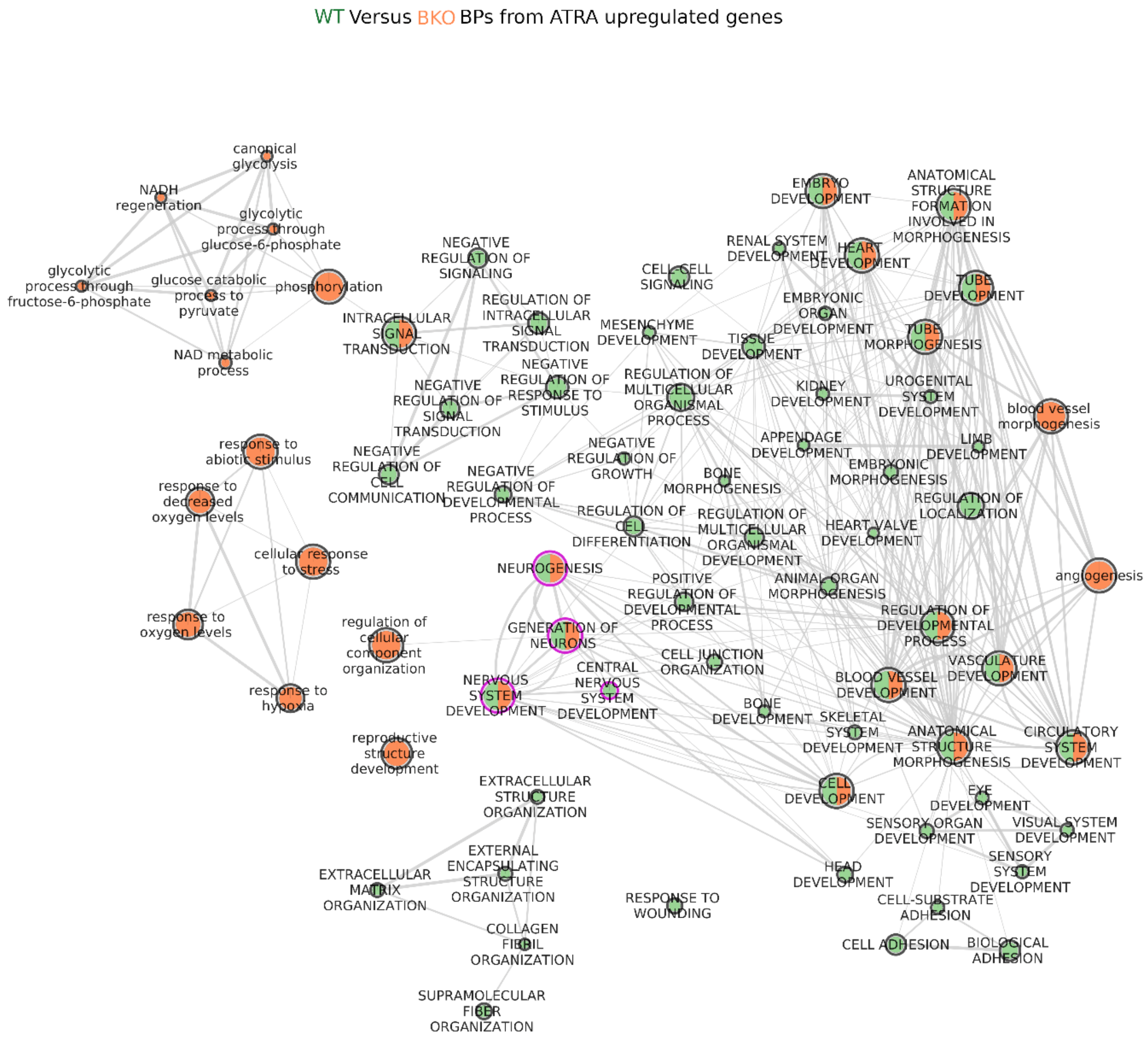

Figure S9. Functional interaction network of GO:Biological Process terms associated with genes upregulated >1.5 X by ATRA treatment (24hr) contrasting differences between in WT (green) and BKO98 cells (Orange). Terms related primarily with neuronal development are circled in purple.

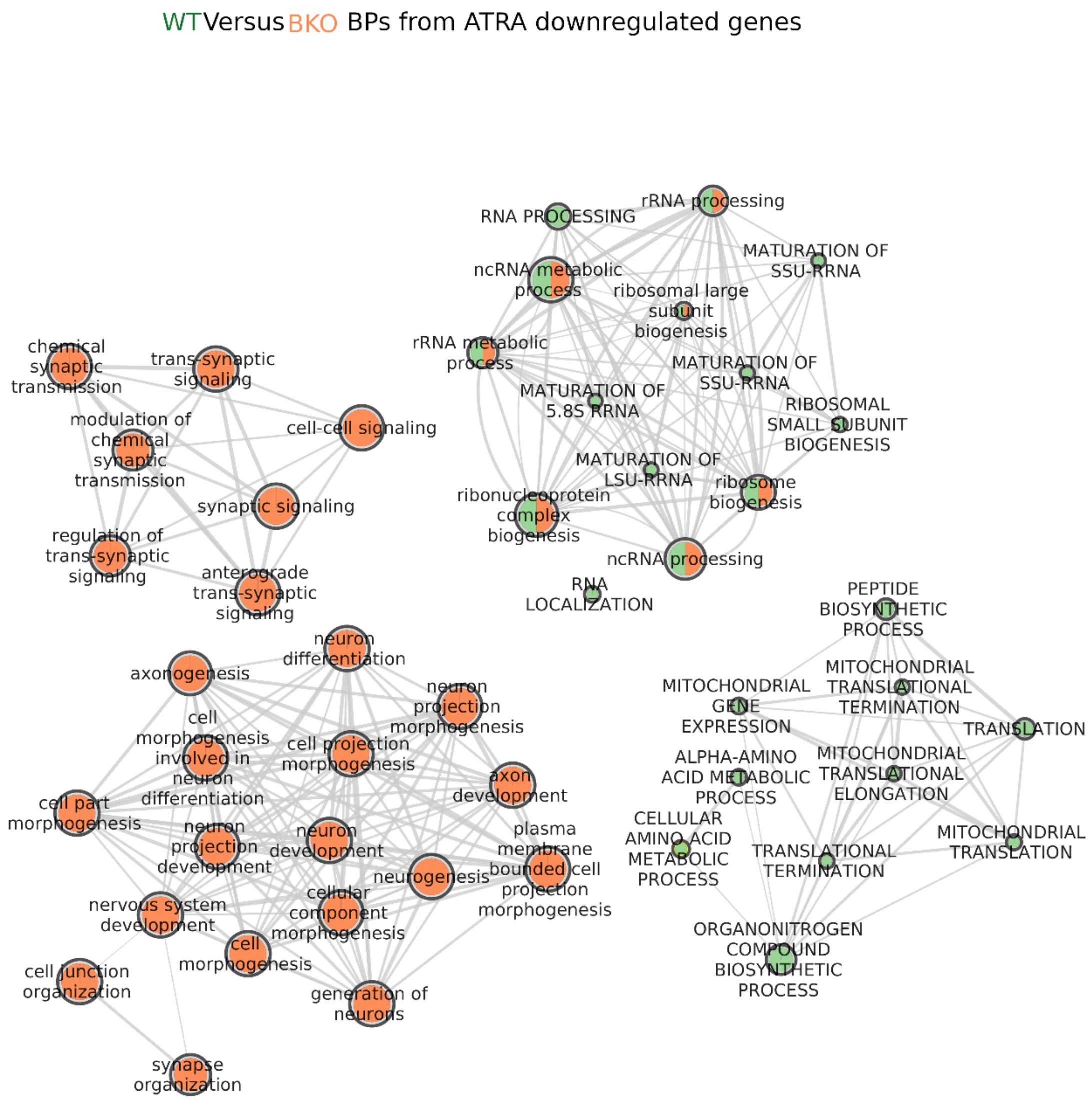

Figure S10. Functional interaction network of GO:Biological Process terms associated with genes down-regulated >1.5 X by ATRA treatment (24hr) contrasting differences between in WT (green) and BKO98 cells (Orange).

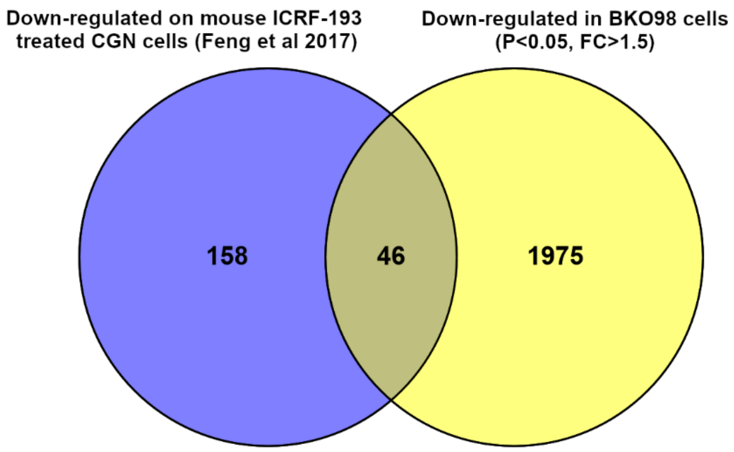

Fig. S11 Overlap between protein coding genes downregulated in TOP2B null BKO98 cells (compared to WT SH-SY5Y cells) and corresponding genes downregulated in ICRF-193 treated mouse CGN cells.

Table S1. Gene symbols and corresponding gene/protein names and relevant details of genes mentioned in the text.

WT SH-SY5Y versus BKO98 (WT_C V BKO_C)		
**Gene Symbol**	**Gene / protein names**	**Comments**
ALK	ALK Receptor Tyrosine Kinase, Anaplastic Lymphoma Kinase	Gain of function mutations associated with neuroblastoma
GABRB3	Gamma-Aminobutyric Acid Type A Receptor Subunit Beta3	Component of the multi-subunit receptor for the inhibitory neurotransmitter GABA
GAP43	Growth Associated Protein 43, Axonal Membrane Protein GAP-43	Expressed at high levels in neuronal growth cones during development and axonal regeneration.
GRIK3	Glutamate Receptor Ionotropic, Kainate 3	Associated with schizophrenia, glutamate receptors are the major excitatory neurotransmitter receptors in the brain
KCNQ3	Potassium Voltage-Gated Channel Subfamily Q Member 3	Functions associated with neuronal excitability
KCNT1	Potassium Sodium-Activated Channel Subfamily T Member 1	
LRRTM2	Leucine Rich Repeat Transmembrane Neuronal 2	involved in excitatory synapse development and maintenance
MAP2	Microtubule Associated Protein 2	Probably involved in forming and maintaining dendrites
NGFR	Nerve Growth Factor Receptor, P75NTR, P75	
NNAT	Neuronatin, Peg5	Possibly involved in the regulation of ion channels during brain development
NOTCH2	Notch Receptor 2, Neurogenic Locus Notch Homolog Protein 2	
NTRK2	Neurotrophic Receptor Tyrosine Kinase 2, TrkB Tyrosine Kinase	Receptor for BDNF (brain-derived neurotrophic factor neurotrophin-4). Receptor tyrosine kinase involved in development and the maturation of the central and the peripheral nervous systems. Modulates neuron survival, proliferation, migration, differentiation, and synapse formation and plasticity.
SEZ6L	Seizure Related 6 Homolog Like	Associated with autism spectrum disorder and may contribute to specialized neuronal endoplasmic reticulum functions.
SRRM4	Serine/Arginine Repetitive Matrix 4,	Splicing factor required for neural differentiation, stimulates alternative splicing and inclusion of neural-specific exons in mRNAs.
VIM	Vimentin	Class-III intermediate filament expressed in various non-epithelial cells, especially mesenchymal cells.

Table 1. ctd

WT SH-SY5Y control versus WT SH-SY5Y + ATRA (WT_C V WT_R)		
**Gene Symbol**	**Gene / protein names**	**Comments**
BCL2	BCL2 Apoptosis Regulator	Suppresses apoptosis in various cell systems including neural cells by controlling the mitochondrial membrane permeability.
CRABP2	Cellular Retinoic Acid Binding Protein 2	Cytosol-to-nuclear shuttling protein facilitating RA binding to its cognate receptor complex
CYP26A1	Cytochrome P450 Family 26 Subfamily A Member 1, P450RAI	Monooxygenase involved in metabolism and synthesis of cholesterol, steroids and other lipids, regulates the cellular level of retinoic acid
CYP26B1	Cytochrome P450 Family 26 Subfamily B Member 1, P450RAI2	see above
DHRS3	Dehydrogenase/Reductase 3, Retinol Dehydrogenase 17	Catalyses the oxidation/reduction of a number of substrates, including retinoids
HEY1	Hes Related Family BHLH Transcription Factor With YRPW Motif 1	Transcriptional repressor, downstream effector of Notch signalling, may promote maintenance of neuronal precursor cells.
HOXC4	Homeobox C4	Transcription factor with a role in morphogenesis
HOXD10	Homeobox D10	Transcription factor with a role in morphogenesis
HOXD8	Homeobox D8	Transcription factor with a role in morphogenesis
MYC	MYC Proto-Oncogene, BHLH Transcription Factor	Transcription factor that plays a role in cell cycle progression, apoptosis, and cellular transformation.
PPARG	Peroxisome Proliferator Activated Receptor Gamma, NR1C3	Forms a heterodimer with retinoid X receptor, involved in adipocyte differentiation
RARB	Retinoic Acid Receptor Beta,	Member of the thyroid-steroid hormone nuclear receptor superfamily, binds retinoic acid mediating cellular signalling in embryonic development, cell growth and differentiation.
RELN	Reelin, RL	Encodes a large extracellular matrix protein involved in cell positioning and neuronal migration during brain development. Associated with schizophrenia, ASD and other neurodevelopmental conditions.
RET	RET Receptor Tyrosine Kinase	Transmembrane receptor tyrosine kinase binding ligands including GDNF (glial cell-line derived neurotrophic factor). Facilitates development of the nervous system and of tissues derived from the neural crest

Table 1. ctd

Long genes		
**Gene Symbol**	**Gene/protein name**	**Comments**
ANK2	Ankyrin 2, brain ankyrin	
CACNA1C	Calcium Voltage-Gated Channel Subunit Alpha1 C	
CNTN4	Contactin 4, Neural Cell Adhesion Protein BIG-2	A phosphatidylethanolamine-anchored neuronal membrane protein, axonal-associated cell adhesion molecule with likely role in neuronal network formation and plasticity. Associated with autism spectrum disorder
CNTN5	Contactin 5	Mediate cell surface interactions during nervous system development.
CNTNAP2	Contactin Associated Protein 2	Member of the neurexin family of nervous system cell adhesion molecules and receptors. Associated with autistic spectrum disorder
DPP6	Dipeptidyl Peptidase Like 6	Single-pass membrane protein peptidase, binds and alters the activity of specific voltage-gated potassium channels.
DSCAM	Down Syndrome Cell Adhesion Molecule	Mediates neuronal cell guidance, receptor for netrin required for axon guidance.
EPHA6	EPH Receptor A6	Receptor tyrosine kinase involved in bidirectional signalling into neighbouring cells. Originally identified as mediators of axon guidance
FHIT	Fragile Histidine Triad Diadenosine Triphosphatase, AP3Ase, FRA3B	
GRID1	Glutamate Ionotropic Receptor Delta Type Subunit 1	Glutamate receptors are the major excitatory neurotransmitter receptors in the brain
GRID2	Glutamate Ionotropic Receptor Delta Type Subunit 2	
GRM1	Glutamate Metabotropic Receptor 1	
GRM5	Glutamate Metabotropic Receptor 5, Protein Phosphatase 1, Regulatory Subunit 86	Activates a phosphatidylinositol-calcium second messenger system, may be involved in the regulation of neural network activity and synaptic plasticity.
KALRN	Kalirin RhoGEF Kinase, Huntingtin-Associated Protein-Interacting Protein	Associated with schizophrenia. Interacts with the huntingtin-associated protein 1,

KCNMA1	Potassium Calcium-Activated Channel Subfamily M Alpha 1	
LRRC1	Leucine Rich Repeat Containing 1, LANO	
NBEA	Neurobeachin, LYST2, Lysosomal-Trafficking Regulator 2	May be associated with autism spectrum disorder
NRG3	Neuregulin 3	Associated with schizophrenia
PCDH15	Protocadherin Related 15, CDHR15, DFNB23	Mediates calcium-dependent cell-cell adhesion, required for maintenance of retinal and cochlear function
PLCL1	Phospholipase C Like 1	
PTPRK	Protein Tyrosine Phosphatase Receptor Type K, Phosphatase Kappa	
PTPRT	Protein Tyrosine Phosphatase Receptor Type T	Associated with autism spectrum disorder and schizophrenia
RALYL	RALY RNA Binding Protein Like, HNRPCL3	
ROBO2	Roundabout Guidance Receptor 2, SAX3 3	Transmembrane receptor for SLIT2, functions in axon guidance and cell migration
SH3GL2	SH3 Domain Containing GRB2 Like 2, Endophilin A1	SH3 Domain Containing GRB2 Like 2, Endophilin A1, associated with late onset Parkinson’s disease

Adrenergic versus Mesenchymal switch

**Gene Symbol**	**Gene/protein name**	**Comments**
ASCL1	Achaete-Scute Family BHLH Transcription Factor 1, HASH1, MASH1	BHLH transcription factor, plays a role in the neuronal commitment and differentiation
DBH	Dopamine Beta-Hydroxylase	Present in adrenal neurosecretory vesicles and chromaffin granules, catalyses the conversion of dopamine to norepinephrine, which is the main neurotransmitter of the sympathetic nervous system and a hormone.
EYA1	EYA Transcriptional Coactivator And Phosphatase 1, Eyes Absent Homolog 1	
FN1	Fibronextin 1	Involved in cell adhesion and migration
FOSL	FOS Like 2, AP-1 Transcription Factor Subunit, FRA2	AP-1 transcription factor family member
GATA2	GATA Binding Protein 2, Endothelial Transcription Factor GATA-2	Member of the GATA family of zinc-finger transcription factors

GATA3	GATA Binding Protein 3	Member of the GATA family of zinc-finger transcription factors
HAND1	Heart And Neural Crest Derivatives Expressed 1, BHLHa27	Member of the basic helix-loop-helix family of transcription factors.
HAND2	Heart And Neural Crest Derivatives Expressed 2, BHLHa26	Member of the basic helix-loop-helix family of transcription factors.
HES1	Hes Family BHLH Transcription Factor 1, BHLHb39, Hairy/Enhancer Of Split 1	Member of the basic helix-loop-helix family of transcription factors, transcriptional repressor of genes activated by other bHLH factors
ISL1	ISL LIM Homeobox 1, Insulin Gene Enhancer Protein ISL-1	Member of the LIM/homeodomain family of transcription factors
JAG1	Jagged Canonical Notch Ligand 1, Jagged 1	Ligand for NOTCH1
KLF13	Kruppel Like Factor 13	Zinc finger transcription factor, associated with schizophrenia
KLF7	Kruppel Like Factor 7	Zinc finger transcription factor
MAML2	Mastermind Like Transcriptional Coactivator 2	Transcriptional coactivator for NOTCH proteins.
NEFL	Neurofilament Light Chain	Neurofilaments are neuronal intermediate filaments, helping maintain neuronal shaper, may also be involved in intracellular transport to axons and dendrites
NEUROG2	Neurogenin 2, NGN2	BHLH transcription factor involved in neuronal commitment and differentiation
NOTCH1	Notch Receptor 1, Neurogenic Locus Notch Homolog Protein 1	
NOTCH3	Notch Receptor 3, Neurogenic Locus Notch Homolog Protein 3	
PHOX2A	Paired Like Homeobox 2A, Paired Mesoderm Homeobox Protein 2A	Transcription factor involved in development of the autonomic nervous system, regulates the expression of tyrosine hydroxylase (TH) and DBH, both essential for the differentiation and maintenance of the noradrenergic neurotransmitter phenotype.
PHOX2B	Paired Like Homeobox 2B, Neuroblastoma Paired-Type Homeobox Protein	Transcription factor involved in development of the autonomic nervous system
SNAI2	Snail Family Transcriptional Repressor 2	Member of the Snail family of C2H2-type zinc finger transcription factors, involved in epithelial-mesenchymal transitions
SYN3	Synapsin III	May be involved in the regulation of neurotransmitter release and synaptogenesis

TH	Tyrosine Hydroxylase	Key role in adrenergic neurones, involved in the conversion of tyrosine to dopamine which is the rate limiting step in the generation of catecholamines.
WWTR1	WW Domain Containing Transcription Regulator 1	Transcription factor, works in conjunction with YAP1, promotes epithelial-mesenchymal transition
YAP1	Yes1 Associated Transcriptional Regulator	Transcription factor, downstream target of Hippo pathway
ZNF356	CDKN1A Interacting Zinc Finger Protein 1, CIZ1, NUP94	

Supplementary Tables

Table S2 Differential gene expression of genes from the REACTOME Signalling by Retinoic Acid pathway. DEG data was filtered to only include values there the Log2FC >1 or <-1, with a Padj value <0.05. RARE score was derived from the presence of a clear RXRA ChIP-seq peak in or near the gene promoter (P), or potential enhancer (presence of RXRA peak coincident with H3K27Ac peak internal (IE), upstream (UE) or downstream (DE) of gene. RAX ChIP-seq data was derived from SK-N-SH cells (ENCODE).

	WT con v WT ATRA		BKO con v BKO ATRA		WT con v BKO con		WT ATRA v BKO ATRA		
**Gene**	Log2FC	Log10(Padj)	Log2FC	Log10(Padj)	Log2FC	Log10(Padj)	Log2FC	Log10(Padj)	**RARE**
CYP26A1	13.5	-112.1	13.9	-40.8			-1.3	-104.5	YES - P
CYP26B1	15.0	-125.7	11.8	-180.5			-2.4	-177.9	YES-UE
CRABP2	5.9	< -255	6.1	< -255	-3.3	-146.1	-3.1	-209.6	YES-UE
DHRS3	9.2	-119.1	7.4	-16.8	-2.4	-1.4	-4.1	-224.0	YES-P/UE
RARA	1.7	-43.1	1.7	-43.5					YES-P
RARB	4.8	< -255	4.1	< -255					YES-P
RARG	1.1	-39.8			1.1	-42.0			YES-P
PPARD			1.1	-18.9					YES-IE
PDK1			1.5	-39.2					NO
PDK3			1.2	-20.7					NO
PDK4	1.2	-2.2			2.7	-13.8	1.7	-7.0	YES-EU
RDH10	1.6	-5.7	2.0	-8.2					NO
AKR1C3	2.6	-5.5			1.3	-1.4			NO
RET	3.8	< -255	3.5	< -255					YES-EU

Table S3. Differential gene expression of selected neuronal differentiation genes. See legend to Table S2

	WT con v WT ATRA		BKO con v BKO ATRA		WT con v BKO con		WT ATRA v BKO ATRA		
**Gene**	Log2FC	Log10(Padj)	Log2FC	Log10(Padj)	Log2FC	Log10(Padj)	Log2FC	Log10(Padj)	**RARE**
NTRK2	7.7	< -255	7.3	-47.3	-3.2	-8.5	-3.6	< -255	NO
BCL2	1.1	-31.3					-2.2	-117.7	YES-IE
NTRK1	1.6	-20.8					-1.2	-11.0	NO
NCAM2	2.8	-46.2			2.6	-39.2			NO
HOXD11	2.1	-128.2	1.3	-50.1					YES - E
HOXD12	3.8	-2.7							YES - E
GAP43					-2.1	-245.1	-2.5		YES-UE
MAP2					-1.9	-92.8	-1.8	-90.1	YES-P/E
SYP							-1.4	-79.5	NO
RELN	-2.8		-1.3	-10.4	-0.9	-6	2.4	-32.1	NO
ARX	-7.3				-7.1	-2.2			NO
SEZ6L			-1.5	-6.3	-6.7	< -255	-7.5	-217.7	NO
NEFM					-1.5	-97.3	-2.4	-242.1	NO
ASCL1	-3.8	-270	-1.0	-13	-3.8	-262			NO
NEUROG2	-1.5	-51			-3.3	-146	-1.1	-19	NO
NEUROD1			-1.6	-23.0					NO
MYC	-4.2		-2.7	-224.5			2.1	-95.9	NO
CDKN1A	1.1		-1.1	-39.6	1.6	-84.9			Yes - P
CCNA1	2.5		2.2	-25.0			-1.2	-13.5	NO

Table S4. KEGG pathways associated with genes down-regulated in TOP2B null cells (BKO98) compared to WT SH-Sy5Y cells.

		Down regulated genes		
**KEGG pathway**	**KEGG id**	**Negative Log10(Padj)**	**term_size**	**intersection_size**
cAMP signaling pathway	KEGG:04024	4.556440493	216	47
Axon guidance	KEGG:04360	4.556440493	181	42
Cholinergic synapse	KEGG:04725	3.360475828	113	28
Dopaminergic synapse	KEGG:04728	3.192431946	131	30
GABAergic synapse	KEGG:04727	3.192431946	89	23
Cocaine addiction	KEGG:05030	3.192431946	49	16
Morphine addiction	KEGG:05032	3.192431946	89	23
Nicotine addiction	KEGG:05033	3.156957993	40	14
Neuroactive ligand-receptor interaction	KEGG:04080	3.001747619	340	58
Glutamatergic synapse	KEGG:04724	2.861574871	114	26

References for Supplemental Figures.

1. Thorvaldsdottir H, Robinson JT, Mesirov JP (2013) Integrative Genomics Viewer (IGV): high-performance genomics data visualization and exploration. Brief Bioinform 14 (2):178-192. doi:10.1093/bib/bbs017

2. Zimmerman MW, Liu Y, He S, Durbin AD, Abraham BJ, Easton J, Shao Y, Xu B, Zhu S, Zhang X, Li Z, Weichert-Leahey N, Young RA, Zhang J, Look AT (2018) MYC Drives a Subset of High-Risk Pediatric Neuroblastomas and Is Activated through Mechanisms Including Enhancer Hijacking and Focal Enhancer Amplification. Cancer Discov 8 (3):320-335. doi:10.1158/2159-8290.CD-17-0993

3. Gartlgruber M, Sharma AK, Quintero A, Dreidax D, Jansky S, Park Y-G, Kreth S, Meder J, Doncevic D, Saary P, Toprak UH, Ishaque N, Afanasyeva E, Wecht E, Koster J, Versteeg R, Grünewald TGP, Jones DTW, Pfister SM, Henrich K-O, van Nes J, Herrmann C, Westermann F (2021) Super enhancers define regulatory subtypes and cell identity in neuroblastoma. Nature Cancer 2 (1):114-128. doi:10.1038/s43018-020-00145-w

4. van Groningen T, Akogul N, Westerhout EM, Chan A, Hasselt NE, Zwijnenburg DA, Broekmans M, Stroeken P, Haneveld F, Hooijer GKJ, Savci-Heijink CD, Lakeman A, Volckmann R, van Sluis P, Valentijn LJ, Koster J, Versteeg R, van Nes J (2019) A NOTCH feed-forward loop drives reprogramming from adrenergic to mesenchymal state in neuroblastoma. Nat Commun 10 (1):1530. doi:10.1038/s41467-019-09470-w

5. van Groningen T, Koster J, Valentijn LJ, Zwijnenburg DA, Akogul N, Hasselt NE, Broekmans M, Haneveld F, Nowakowska NE, Bras J, van Noesel CJM, Jongejan A, van Kampen AH, Koster L, Baas F, van Dijk-Kerkhoven L, Huizer-Smit M, Lecca MC, Chan A, Lakeman A, Molenaar P, Volckmann R, Westerhout EM, Hamdi M, van Sluis PG, Ebus ME, Molenaar JJ, Tytgat GA, Westerman BA, van Nes J, Versteeg R (2017) Neuroblastoma is composed of two super-enhancer-associated differentiation states. Nat Genet 49 (8):1261-1266. doi:10.1038/ng.3899

6. Gonzalez-Buendia E, Zhao J, Wang L, Mukherjee S, Zhang D, Arrieta VA, Feldstein E, Kane JR, Kang SJ, Lee-Chang C, Mahajan A, Chen L, Realubit R, Karan C, Magnuson L, Horbinski C, Marshall SA, Sarkaria JN, Mohyeldin A, Nakano I, Bansal M, James CD, Brat DJ, Ahmed A, Canoll P, Rabadan R, Shilatifard A, Sonabend AM (2021) TOP2B Enzymatic Activity on Promoters and Introns Modulates Multiple Oncogenes in Human Gliomas. Clin Cancer Res 27 (20):5669-5680. doi:10.1158/1078-0432.CCR-21-0312
